# Structural basis of aquaporin-4 autoantibody binding in neuromyelitis optica

**DOI:** 10.1126/sciadv.adq7560

**Published:** 2025-02-21

**Authors:** Meghna Gupta, Nitesh Kumar Khandelwal, Andrew Nelson, Peter Hwang, Sergei Pourmal, Jeffrey L. Bennett, Robert M. Stroud

**Affiliations:** ^1^ Department of Biochemistry and Biophysics, University of California San Francisco, San Francisco, CA 94143, USA.; ^2^ Department of Chemical Physiology and Biochemistry, Oregon Health & Science University, Portland, OR 97239, USA.; ^3^ Departments of Neurology and Ophthalmology, Programs in Neuroscience and Immunology, University of Colorado School of Medicine, Anschutz Medical Campus, Aurora, CO 80045, USA.

## Abstract

Neuromyelitis optica (NMO) is an autoimmune disease of the central nervous system where pathogenic autoantibodies target the water channel aquaporin-4 on human astrocytes causing neurological impairment. Autoantibody binding leads to complement-dependent and complement-independent cytotoxicity, ultimately resulting in astrocyte death, demyelination, and neuronal loss. Aquaporin-4 assembles in astrocyte plasma membranes as symmetric tetramers or as arrays of tetramers. We report molecular structures of aquaporin-4 alone and bound to Fab fragments from patient-derived NMO autoantibodies using cryogenic electron microscopy. Each antibody binds to epitopes comprised of three extracellular loops of aquaporin-4 with contributions from multiple molecules in the assembly. The structures distinguish between antibodies that bind to the tetrameric form of aquaporin-4 and those targeting higher-order orthogonal arrays of tetramers that provide more diverse bridging epitopes.

## Teaser

Pathogenic autoantibodies in neuromyelitis optica bind three multiply presented loops on arrays of human aquaporin-4.

## INTRODUCTION

Neuromyelitis optica (NMO) is a severe and debilitating autoimmune disease of the central nervous system (CNS). In most cases (≥80%), NMO is mediated by antibodies that target the extracellular loops of the human water channel aquaporin-4 (AQP4) in the plasma membrane (*1*). Pathogenic AQP4 immunoglobulin G autoantibodies (AQP4-IgGs), when they interact with AQP4 on astrocytes of the CNS, initiate tissue injury and cause neurological impairment through both lytic and nonlytic mechanisms (*2*). AQP4-IgG–mediated effector functions, complement-mediated cytotoxicity (CDC), and antibody-dependent cell-mediated cytotoxicity cause targeted astrocyte lysis, promote immune cell infiltration, and drive demyelination, axonal injury, and neuronal destruction (*3*–*7*). CDC activation is due to binding of several IgGs into a multimeric platform for C1q assembly (*8*). Current therapies are based on strategies that may result in substantial short- and long-term adverse events (*9*). To advance NMO therapy and diagnosis, we sought to define the structural basis of autoantibody binding, the initial event of the disease, using patient-derived AQP4-specific recombinant antibodies (rAbs).

The epitopes in NMO are formed by three well-ordered extracellular loops (loops A, C, and E) displayed on each AQP4 molecule. Four monomeric water channels form symmetric tetramers, as in all aquaporins, and therefore, the conformational epitopes can include the same extracellular loops from the four adjacent monomers. A higher-order assembly of AQP4 is formed by a splice variant with a start site at Met^23^ (M23). M23 isoform lacks the first 22 amino acids as compared to the full-length AQP4 (M1) on the cytoplasmic side of the plasma membrane. M23 preferentially forms two-dimensional (2D) orthogonal arrays of particles (OAPs) of tetrameric AQP4 and displays the identical extracellular epitopes as the tetramers. M1 disfavors OAP formation, in part due to palmitoylation at Cys^13^ and Cys^17^ on the cytoplasmic side. Therefore, OAPs present additional copies of the epitopes composed of A, C, and E loops arrayed different orientations by adjacent tetramers within the OAPs. The distribution of M1 to M23 is determined by their relative expression and a combination of post-translational modifications (*10*). Higher proportion of M1 isoform limits the size of OAPs, suggesting that M1 is incorporated into the lattice and limits its extent (*10*).

Patient-derived anti-AQP4 rAbs do not recognize linear peptide epitopes that correspond to the loops A, C, or E, indicating that combinations of the 3D structures of these loops displayed on the external surface of AQP4 are the basis of the conformational epitopes (*11*, *12*). In cell-based assays, a subset of purified AQP4-IgGs bind equally well to M1 as to M23 AQP4, indicating that their epitopes are contained within the AQP4 tetramer. However, many AQP4 rAbs have higher affinities for M23 OAPs. These factors imply that these AQP4 rAbs bind to conformational epitopes composed of multiple interactions from neighboring tetramers. The comparison of Fab fragment and divalent IgG binding in these assays did not show significant difference in their binding affinities, suggesting that each IgG binds the target with one Fab fragment, without any avidity effect of bivalent binding by IgGs (*13*). Mutagenesis and changing M1:M23 expression ratios demonstrate that the higher affinity of Fab binding is due to OAP formation. Here, we determined the structural basis of binding of human AQP4 to the patient-derived AQP4-rAb Fab fragments from (i) rAB58, which binds equally well to both M1 and M23 AQP4 isoforms, and (ii) rAB186, which binds ~55 times better (lower *K*_d_) to the M23 OAPs than to M1 (*13*).

## RESULTS

### Cryo-EM structure of human AQP4 in lipid nanodiscs

**Fig. 1.**
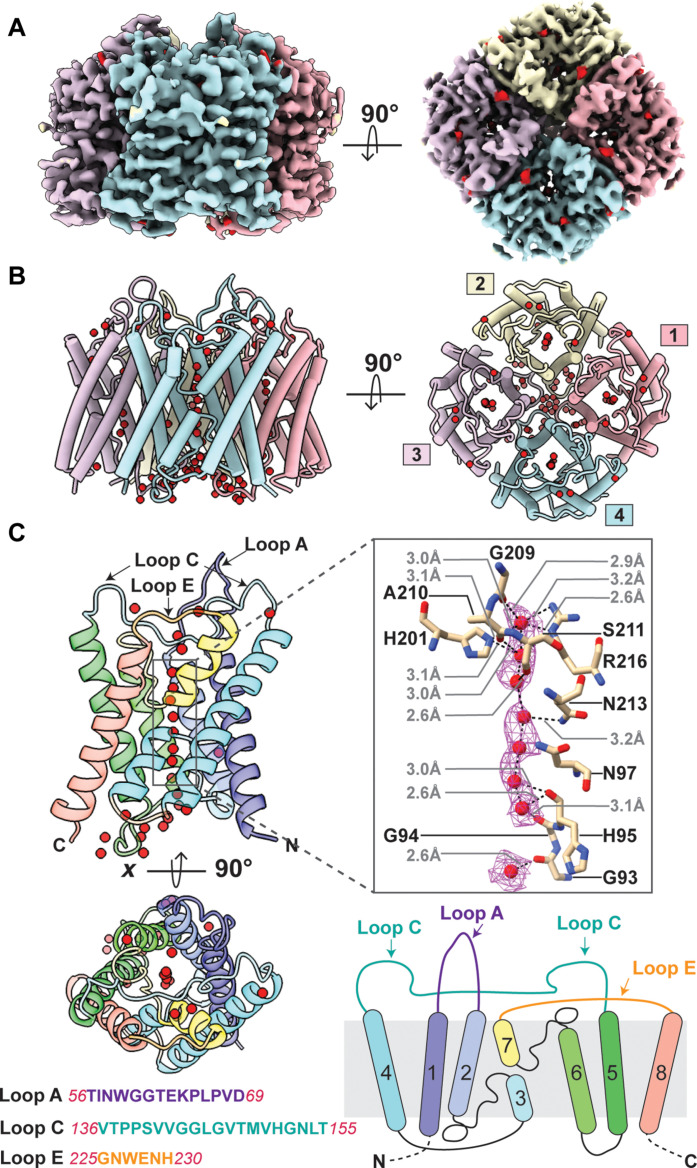
Structure of human AQP4 tetramer in nanodiscs. (**A**) AQP4 density map determined by cryo-EM at 2.1-Å resolution, side view and top view showing the extracellular surface of the same. Each monomer is colored differently, blue, pink, and lemon, thistle counterclockwise. The four individual water channels contain water molecules (shown in red). (**B**) Side view and top view cartoon of the AQP4 tetramer model built using the density map in (A). The monomers are colored as (A) and numbered counterclockwise. (**C**) AQP4 monomer shown in rainbow (from N to C terminus) in the side and top views representing location of water molecules. Distances listed in gray are hydrogen bond distances. These include between main chain hydrogen bond acceptors of carbonyl oxygens of G209 to N213 and the donor oxygen (OH) of water molecules and between the acceptor carbonyls of G93 to H95 and water molecules; hydrogen bond donors between the two side chain NHs of R216; a side chain NH of N213, and N97 to water molecules; and distances between the water molecules as seen in the cryo-EM structure. The three extracellular loops A, C, and E are shown in the side view. Key amino acid residues interacting with the waters are highlighted and experimentally determined waters in the density maps are shown with mesh surface. A cartoon of AQP4 monomer depicting the relative organization of transmembrane helices in rainbow. The sequence of each extracellular loop is shown.

The AQP4 M1 isoform was purified as a homo-tetrameric complex and reconstituted into lipid nanodiscs composed of soybean polar lipid extract surrounded by the membrane scaffold protein (MSP)–1E3D1 (fig. S1). We determined the structure of the AQP4 M1 tetramer to 2.1-Å resolution by cryo–electron microscopy (cryo-EM) (Fig. 1 and figs. S2 and S3). The overall structure, the loops, the water channel, and the continuous line of hydrogen bonded water molecules through it are essentially as in our previously determined crystal structure of AQP4 using x-ray diffraction. The root mean squared deviation (RMSD) at Cα positions is ~0.3 Å for AQP4 cryo-EM versus x-ray diffraction structures (*14*) (fig. S4 and table S1). To obtain the crystal structure of AQP4 tetramers, the protein sample in detergent was treated with trypsin to remove flexible regions of the protein, which then gave a 1.8-Å-resolution electron density map. For the structure reported here, the full-length protein was reconstituted into lipid nanodiscs to maintain a more native environment and was not treated with trypsin. Although the protein was full length, the first 31 amino acid residues at the N terminus and 69 amino acid residues at the C terminus (254 to 323) are not observed in the cryo-EM map, consistent with the flexibility of these cytoplasmic regions on the opposite side to antibody binding.

The structure shows the three extracellular loops to which the AQP4-IgGs bind (*11*, *12*, *15*). Loop A (56-TINWGGTEKPLPVD-69) between transmembrane helices TM1 and TM2 has a well-defined stable structure and extends the farthest, ~21 Å above the plane of the membrane. This loop presents the polar charged side chains of E63 and K64 at the extremity (Fig. 1) and is closest to the fourfold symmetry axis of the tetrameric assembly of AQP4. Loop C (136-VTPPSVVGGLGVTMVHGNLT-155) between TM4 and TM5 is the longest loop of 20 amino acids and traverses the width of AQP4. However, side chains in the center of the loop C (145-LGVT-148) are buried and interlocked in the membrane surface such that there are two distinct epitopes of loop C that are presented: the proximal C loop (136-VTPPSVVGG-144) and the distal C loop epitope (149-MVHGNLT-155). Loop E (225-GNWENH-230) between TM7 and TM8 is the shortest loop, is close to the membrane surface, and is the most distant radially from the fourfold axis of AQP4.

Densities define the line of hydrogen-bonded water molecules throughout the pore of each channel (Fig. 1). Water molecules are hydrogen bonded to side chains of His^201^ and Arg^216^ in the “selectivity filter” of the channel. The central water-polarizing site is formed by hydrogen bond donors from two Asn residues N97 and N213 of the twinned signature sequences of all aquaporins that consist of two -Asn-Pro-Ala- (-NPA-) motifs, one from each half of the sequence. These polarize the central water that positions all waters to orient donor hydrogen bonds outward from the center toward each side preventing any ion, even hydronium from passing through the channel. The fourfold symmetry axis relating the four molecules of the AQP4 tetramer is surrounded by hydrophobic side chains and is not functional for transport.

### Fab binding to AQP4

We focus on understanding the difference between classes of Fabs that bind equally well to M1 tetramers, as to M23 OAP arrays, versus those that bind tighter to OAPs. rAb58 binding is not significantly better to M23 OAPs (*K*_d_ = 68 ± 9 nM) than to M1 tetramers (*K*_d_ = 147 ± 20 nM), while rAb186 binds ~55-fold better to M23 OAPs (*K*_d_ = 146 ± 25 nM) than to M1 tetramers (*K*_d_ = 8100 ± 3000 nM), suggesting additional interaction with neighboring tetramers in OAPs (*16*). Independent of the epitope pattern recognized, some AQP4 rAbs are dependent on residues H151-L154 in distal loop C of AQP4 and demonstrate enhanced CDC (*8*). rAb58 binding is independent of AQP4 H151-L154 residues, while rAb186 interaction depends on H151-L154 residues (*8*). The binding affinity of each Fab for M1 tetrameric AQP4 was confirmed using biolayer interferometry (BLI), and their structures and binding interactions were determined by cryo-EM.

### Fab58 binding to a AQP4 tetramer

**Fig. 2.**
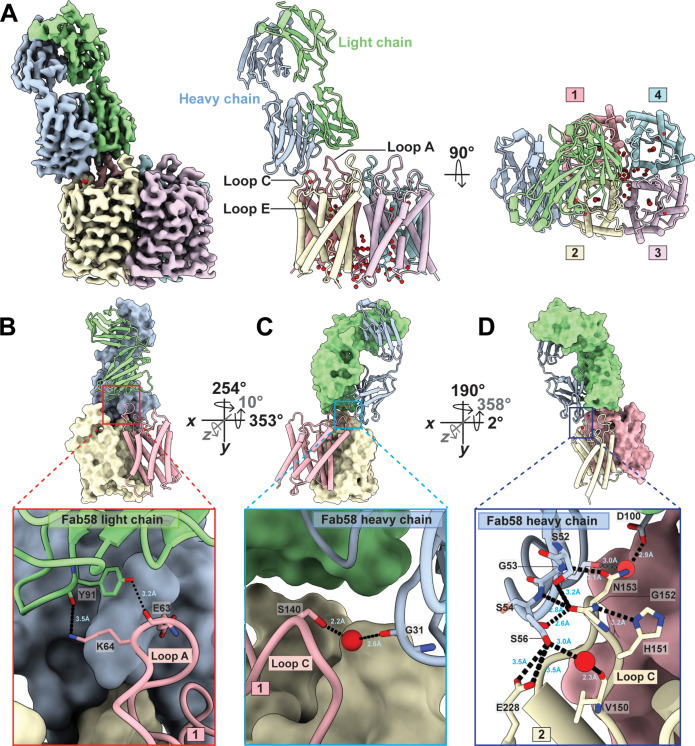
Fab58 binding to human AQP4 M1 tetramer. (**A**) Side view of the cryo-EM density map at 2.5-Å resolution showing AQP4 tetramer and Fab58 interacting with the extracellular loops. Fab58 HC is shown in light steel blue and LC in pale green. A cartoon of the side view and top view of the model built using the density map with the same color scheme and numbered counterclockwise. One molecule of Fab58 makes interactions with two neighboring AQP4 monomers in the tetramer. (**B**) Molecular interactions between the LC of Fab58 and loop A of monomer 1 of AQP4 tetramer. Hydrogen bonds are depicted with a black dotted line and hydrogen bond lengths between atoms in Å are in blue. (**C**) Molecular interactions between the HC of Fab58 and loop C of monomer 1 of AQP4 tetramer mediated by a water molecule. (**D**) Molecular interactions between the HC of Fab58 and AQP4 loop C of monomer 2 of AQP4 tetramer. Two water molecules in red play an important role in this interaction.

2D class averages of cryo-EM images show that each AQP4 tetramer is bound by only a single Fab58 (Fig. 2 and fig. S5). The 3D structure obtained at 2.5-Å resolution shows that one Fab58 binds a compound epitope between two adjacent monomers within a single tetramer (Fig. 2 and figs. S5 and S6). The binding of one Fab58 sterically overlaps the binding sites for Fab58s to the neighboring monomers on either side within the same tetramer (Fig. 2). The van der Waals surface of Fab58 extends to the central fourfold axis. rAb58 binds equally well to tetramers as to OAPs in a cell-based assay with *K*_d_ values reported between ~60 and 150 nM (*8*, *16*). The Cα RMSD value for the overall structure comparing AQP4 apo and AQP4-Fab58 is 0.171 Å, while that for loop A is 0.347 Å, for loop C is 0.201 Å, and for loop E is 0.148 Å (fig. S7 and table S1). There is no structural impediment to water access to the channel entrance from the presence of bound Fab58 as established quantitatively using the HOLE program (*17*) (Fig. 2 and fig. S8).

Fab58 contacts residues of loop A and the proximal end of loop C from one AQP4 monomer and the distal end of loop C from its neighboring monomer as seen counterclockwise from the external side of the cell in the tetramer (Fig. 2). Y91 of Fab58 light chain (LC) binds amino acids 63-EK-64 of loop A, while the carbonyl of G31 of Fab58 heavy chain (HC) interacts with loop C amino acid S140 via a water molecule (Fig. 2, B and C, and fig. S9). Fab58 HC provides a polar environment and makes hydrogen bonds from the N-H of G53 and D100 HC via water to N153, and three hydrogen bonds to the carbonyl of G152 of distal loop C from the adjacent monomer. S56 HC makes a hydrogen bond via a water molecule to the carbonyl of V150 (loop C) and to the carboxyl group of E228 (loop E) (Fig. 2D).

The side chains of two aromatic rings W32 in LC and Y101 in HC of Fab58 sandwich the aliphatic K64 side chain in the “-EKP-” segment of loop A. These residues on the Fab are each buttressed by other interactions within the Fab58 R30 in LC and D100 in HC, suggesting that this pi-aliphatic cation-pi interaction can be very favorable for antigen binding. The terminal NH_3_^+^ of K64 is hydrogen bonded to the carbonyl of Y91, interactions that together can fix the aliphatic chain of K64 and then account for a large component of the association energy of Fab58 to AQP4 (fig. S9). The Fab58 residues participating in AQP4 binding are restricted to the complementarity determining regions as summarized in fig. S10.

Substitutions of certain residues in the extracellular loops of AQP4 to alanine diminish rAb58 binding as measured by fluorescence-based imaging, supporting a role in target sites bound by Fab58 (*12*). Neither H151 nor L154 is close to the interaction interface and hence not part of the epitope for Fab58. Mutation K64A, which removes the aliphatic side chain, reduces rAb58 binding by ~80% the largest amount when compared with mutations in nearby residues from T62 to V68 (-TEKPLPV-) (*12*). In summary, rAb58 binds with similar affinity to M1 tetramers as to M23 OAPs since the compound epitope overlaps loops A and C from two adjacent monomers within a single tetramer of AQP4 (Fig. 2) (*13*).

### Interaction of multiple Fab186 molecules to a AQP4 tetramer

**Fig. 3.**
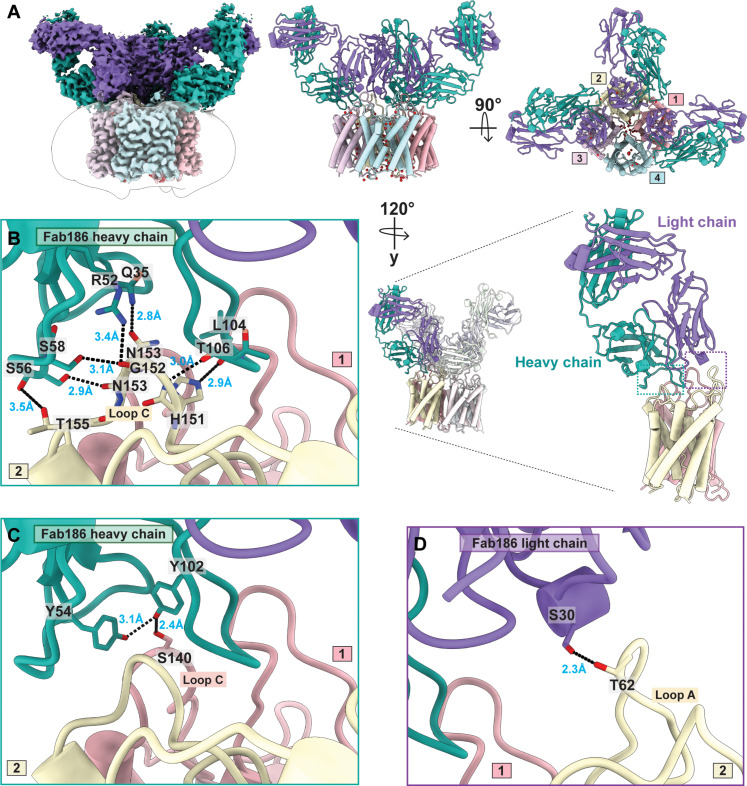
Fab186 binding to human AQP4 M1 tetramer. (**A**) Side view of the cryo-EM density map surrounded by a detergent micelle at 2.9-Å resolution showing AQP4 tetramer and three Fab186 molecules interacting with the extracellular loops. Fab186 HC is shown in light sea green and LC in medium purple. A cartoon of the side view and top view of the model built using the density map with the same color scheme and numbered counterclockwise. One Fab186 makes interactions with two neighboring AQP4 monomers in the tetramer. (**B**) Molecular interactions between the HC of Fab186 and loop C of monomer 2 of AQP4 tetramer. Bond length in Å is in blue and hydrogen bonds are shown with black dotted line. (**C**) Hydrogen bonded interactions between the HC of Fab186 and loop C of monomer 1 of AQP4 tetramer. (**D**) Hydrogen bonded interactions between the LC of Fab186 and loop A of monomer 2 of AQP4 tetramer.

The cryo-EM structure of Fab186 binding to the M1 tetramers at 2.9-Å resolution shows that the primary contacts are between the Fab186 HC and the distal portion of loop C from one monomer, and the proximal region of loop C from the neighboring monomer clockwise in the tetramer as seen from the extracellular side of the tetramer (Fig. 3 and figs. S11 and S12). N153 in the distal loop C AQP4 is in a central position with supporting interactions from T155 and G152. The HC of Fab186 (Q35, R52, S56, and S58) makes multiple contacts through side chains as well as the main chain (Fig. 3B). T155 and N153 make hydrogen bond with S56 of Fab186 HC; G152 interacts with R52 and S58 of Fab186 HC. The N153 side chain forms an additional interaction with Fab186 HC Q35. Y54 and Y102 of the Fab186 HC clasp S140 of the loop C from the neighboring AQP4 monomer (Fig. 3C). Both distal and proximal loop C from adjacent monomers contribute to this compound epitope. S30 of the Fab186 LC hydrogen bonds with T62 and impinges on E63 of loop A (Fig. 3D). There is no direct interaction with the key residues 63-EKP-65, although mutations in these residues may change the conformation of the loop and orientation of T62 side chain, hence causing sensitivity (*12*). The Cα RMSDs between AQP4-Fab186 versus AQP4 apo are 0.254 Å overall and 0.397, 0.374, and 0.244 Å for loops A, C, and E, respectively, depicting minimal conformation change upon Fab binding (fig. S7 and table S1).

**Fig. 4.**
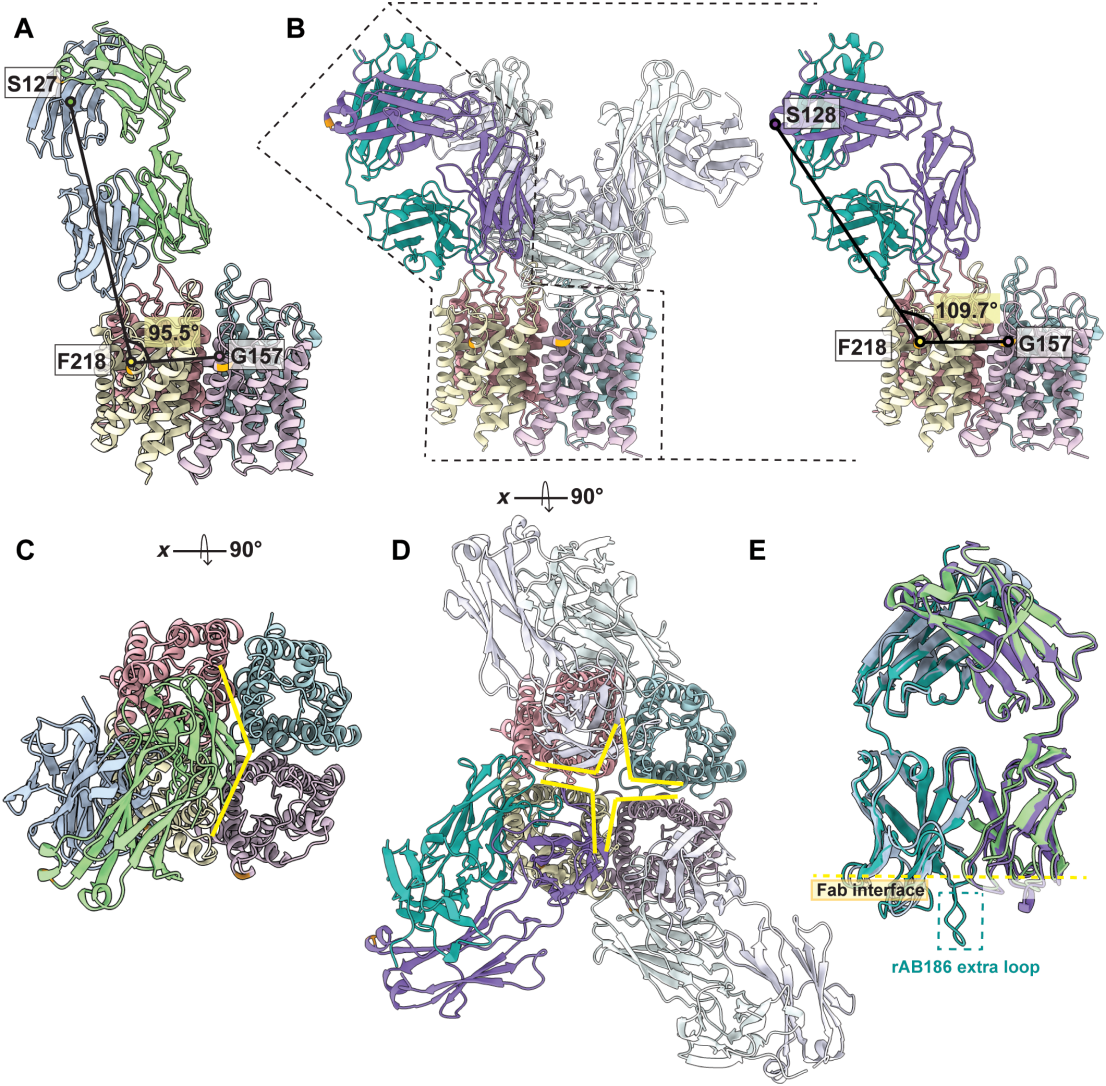
Comparison between Fab58 and Fab186 binding on AQP4 tetramer. (**A**) Fab58 binds on AQP4 surface with an angle of 95.5° between selected labeled Cα atoms, while (**B**) Fab186 binds at 109.7° measured using the orthologous amino acid residues to compare Fab58 and Fab186 binding. (**C**) Occupancy of Fab58 on AQP4 tetramer shown in the top view exhibiting steric limitation to accommodate another Fab58 molecule. (**D**) Fab186 binding at a steeper angle enables efficient binding of four Fabs. We see full density for three molecules of Fab while fourth molecule has a weak density as shown in fig. S9. (**E**) Overlay of Fab58 and Fab186 structures shows their different loops relative to a constant template. Fab186 has extended regions and one of these takes part in key interaction with H151 of loop C differentiating it from Fab58.

Fab186 has two extended loops one from HC, another from the LC. The extended loop from HC engages the distal loop C H151/L154 of AQP4, explaining the sensitivity of Fab186 to mutations in distal C-loop (Fig. 4E) (*8*). L104 and T106 of Fab186 HC directly insert into the AQP4 cleft where H151 is presented on loop C (Fig. 3B). There is no direct interaction with L154 in our structure, emphasizing that mutations as in this residue have indirect effects on nearby conformations of the loop that affect Fab186 binding.

**Fig. 5.**
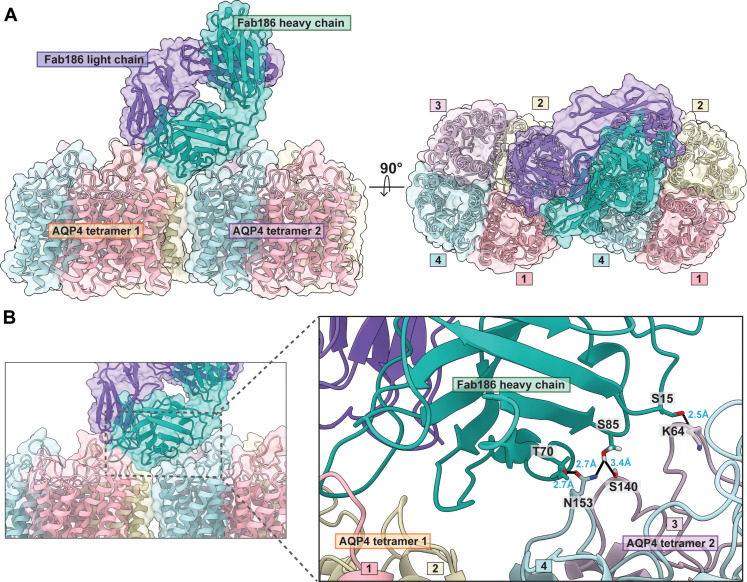
Understanding potential rAB186 association with OAPs. (**A**) Human AQP4 structure superposed on the two neighboring tetramers of rat AQP4 M23 isoform tetramers from the 2D crystal lattice structure based on electron diffraction analysis (PDB ID 2D57). Two of the monomers in one of the tetramers were replaced with a minimal Fab186-bound structure consisting of two monomers of AQP4 and one molecule of Fab186. These space-filling representations emphasize the proximity of the Fab186 to the neighboring tetramer, favored by AQP4 M23 OAPs. The side view and top view exhibiting the possibility of Fab186 interaction spanning two AQP4 tetramers. (**B**) Close-up of the Fab186 HC potential interactions [as in (A)] with loop A and loop C key of the neighboring tetramer.

Cryo-EM images of Fab186-AQP4 show binding of up to four Fabs per AQP4 tetramer (figs. S11 and S13). The fourth Fab is visible only at lower contours of the map, probably due to statistical occupation (fig. S13). Each Fab186 binds, bridging two monomers within the tetramer (Figs. 3 and 4) at an angle away from the fourfold axis, suggesting that the rAb186-AQP4 may also incorporate contacts from neighboring tetramers in the OAPs (Fig. 5). The binding angles and orientation of Fab58 and Fab186 are different, although both are sensitive to mutations in all three loops of AQP4 (Fig. 4). A linear morph between models of Fab58 and Fab186 binding over two AQP4 monomers highlights the differences in interaction (movie S1).

### Modeling the binding of rAb186 to AQP4 OAP arrays

rAb186 binds ~55-fold tighter to M23 OAPs than to M1 tetramers (*13*). The OAPs place multiple tetramers close to each other, such that the intertetrameric conformational epitopes provide more diversity than available within the tetramer. To see which residues of neighboring tetramers in OAPs might interact with rAb186, relative placement of the AQP4 tetramers was modeled on the basis of known structures of OAPs. In vitro, M23 can form double layers of orthogonal arrays that face each other via their extracellular surfaces as determined by electron diffraction analysis (*18*) (fig. S14). Hence, the double layer between the extracellular leaflets results in compaction of the loops A, C, and E, between the interfaces relative to the structures we determine here. Therefore, to model the cellular surface 3D epitopes, we replaced each tetramer in a single layer of the crystal lattice by the M1 tetramer structure (fig. S15). As evidence that the single layer of the double-layered crystal lattices is the physiological lattice, the unit cell dimensions of the arrays in rat M23 AQP4 layers *a* = *b* = 69.0 Å are identical to those of the M23 single layer arrays formed in human OAPs in cells (*18*).

The interactions between tetramers within the bilayer are also highly conserved in other species where lattice formation is also found, although there is no yet recognized function for these OAP lattices. Using a (') to signify an amino acid from an adjacent tetramer in the lattice, specificity of each intertetramer close interaction involves W231-L'160/G'157 and W'231-L160/G157, and of Y250-R'108 and Y'250-R108, and one dimeric pair interaction of I239-I'239 (*18*). G157, W231, and I239 are the hydrophobic residues that constitute the extracellular side of the intertetramer interactions. On the cytoplasmic side, R108 and Y250 stabilize the interface (*18*) (fig. S16). These interactions provide the rationale for the specificity in the tetramer-tetramer interface that drives in-plane lattice formation. These intertetramer residues are all conserved between human, rat, mouse, and bovine, with only one conservative change in mouse from I239 to M239, suggesting some yet undefined physiological role. Therefore, this lattice reflects the single layer OAPs that interact with autoimmune rAbs to augment the immune response and allows us to ask how the rAb186 might gain additional interactions from the neighboring tetramers.

The structure of Fab186 is angled with its major axis ~20° away from perpendicular toward the membrane plane (Fig. 4) and forms close contacts of the HC with two monomers (termed monomers 3 and 4) of a neighboring tetramer in the model OAP (Fig. 5). The observed interaction with the second tetramer shows that the S15, T70, and S85 of Fab186 HC could interact with K64 of loop A and S140 of loop C (monomer 3), and N153 of loop C (monomer 4), of the neighboring tetramer in the OAP array (Fig. 5).

## DISCUSSION

We determined the cryo-EM structure of full-length human M1 (residues 1 to 323) tetrameric AQP4 in lipid nanodiscs to 2.1-Å resolution. When compared with our previous x-ray crystal structure of AQP4 in detergent micelles, determined at 1.8-Å resolution (*14*), the overall RMSDs are within the random deviations expected from refinement at these resolutions, for Cα 0.304 Å, and an all-atom RMSD of 0.93 Å (table S1). These differences may include small local changes due to differences in structure determination procedures. The protein that we crystallized was treated to tryptic proteolysis that removed flexible regions at the N terminus, and C terminus both on the cytoplasmic surface of the protein, to leave residues 20 to 259 in the crystal structure of AQP4. The crystal structure had weak density for residues 21 to 31 and 255 to 259, indicating that 10 residues at the N terminus and 4 at the C terminus are not well ordered and are flexible in the crystal. These same terminal residues 1 to 31 and 254 to 323 are not visible in the cryo-EM structure due to their flexibility. RMSDs listed for loop A are artifactual, due to poor density for three residues at the most distal end of the loop in the x-ray crystal structure. Loop A extends farthest (~21 Å) above the membrane plane and into solvent with a Cα RMSD of 0.674 Å. For loop C, the Cα RMSD is 0.413 Å. Loop E is the most external from the fourfold axis of the AQP4 tetramer and the Cα RMSD is 0.439 Å. It contains two tryptophan side chains in contact with and therefore sensitive to the difference between the nanodisc in cryo-EM and detergent in the crystal structure (fig. S4 and table S1).

The interaction of autoimmune antibodies with their target epitopes raises the question as to whether the antibodies modify the epitopes or are dominated by binding the intact configuration of the target epitopes. In the two cases we describe here, the epitope is remarkably constant in its configuration before and after Fab binding. The interaction of Fab58 or Fab186 autoantibodies with their target epitopes causes little change in AQP4 upon binding. The comparison within the loops A, C, and E in the Fab bound versus apo structures is graphed and in stereoscopic view in fig. S7. The minimal RMSD change upon Fab binding suggests that these antibodies are selected to bind to structured target epitopes with little plastic adaptation of the epitope. The Fabs do so in a manner that gains favorable thermodynamic contributions to binding from shape complementarity, enthalpic terms from compatible hydrogen bonding, and favorable entropic contributions by desolvation, while also avoiding clashes. The cryo-EM data collection and model building statistics are listed in table S2.

AQP4 conducts water at close to the diffusion limit for a pore of this size in response to any osmotic pressure, as evoked by ion conductance during neuronal action (*19*–*21*). The water channel contains a polarized single file of water molecules supported by hydrogen bond acceptor and donors along the entire channel pathway (Fig. 1). The Fab-bound structures we report here do not impinge on the water channel that show ordered water molecules throughout the channel, or access of water to the channels, and thus do not interfere with this function. The similarity in structures is quantitated by calculation of the excluded volume to and through the channels by the program HOLE (*17*) (fig. S8). This is consistent with multiple published studies that demonstrate that IgG binding does not impair water conductance of AQP4 (*15*, *22*, *23*). The extracellular surface epitopes are structured extensions into the extracellular space. Upon binding to the AQP4 epitopes antibody loops add further distance from access to the water channel. AQP4 internalization is most likely to be the operative mechanism contributing to symptoms of NMO (*24*).

The interactions of rAb186 with the second tetramer present in OAPs can account for the observed tighter binding to OAPs with ~55-fold higher affinity than to tetramers of AQP4 (*16*). Using Δ*G*^0^_f_ = RT ln(*K*_d_) = 1.4 log_10_ (55) = −2.4 kcal/mol, this energetic factor can be accounted for by the additional Fab186 interactions with the second, adjacent tetramer in OAPs. There are several additional hydrogen bonds indicated in the OAP model of AQP4-Fab186 (Fig. 5). These are listed in the order Fab186 first, followed by neighboring AQP4 tetramer, and listing distances in the model up to 3.6 Å with reasonable geometry. Hydrogen bonds can form between S85/OG-N153/NH_2_ (2.7 Å) and T70/OG1-N153/OD1 (2.7 Å) of monomer 4. Most other interactions are to loops A S15/OG-K64/NH (2.5 Å) and loop C of monomer 3 S85/OG-S140/OG (3.46 Å) (Fig. 5B). There is only one clash, between the side chains of S15 and E63 at the tip of loop A (monomer 3) that could be alleviated by slight movement of the side chains apart in the complex, with potential of forming productive hydrogen bonds between them. The Cα of S15 and E63 without any adjustment in the model are 2.96 Å apart, an indication of the small adaptation that may occur in the complex.

The reduction in solvent accessible surfaces in the complex can also contribute to entropy gain toward the free energy of interaction. Specifically, the program PISA maps the change in solvent accessible surface and gives an estimate of Δ*G*^0^_f_ = −2.6 kcal/mol for the interface between the HC of Fab186 and the monomer 4 of the “second” tetramer (*25*). The largest contributors from AQP4 are S140 (54.4 Å^2^) and P139 (38.6 Å^2^) of monomer 3. The total buried surface area at the interface is monomer 3 (139 Å^2^) for predicted Δ*G*^0^_f_ = +0.7 kcal/mol and monomer 4 (241.1Å^2^) for predicted Δ*G*^0^_f_ = −2.6 kcal/mol (probably X2) because of both sides.

A third factor in affinity for the neighboring tetramer in OAPs, Fab186, presents an electrostatically positive surface to the interface, reflecting presence of K83 and R68, while AQP4 is predominantly negatively charged in the interacting surface of the second tetramer, reflecting presence of E63 of AQP4. Therefore, the additional binding energy (−2.4 kcal/mol) toward OAPs is easily accounted for with minimal strain in adjacent tetramers, and positive energetic components from hydrogen bonds (~0 to −6 kcal/mol), burial of solvent accessible surfaces (−2.6 kcal/mol), and electrostatic complementarity. Last, the binding of each Fab186 while it allows others to bind within the same tetramer blocks the binding of three potential sites on the neighboring tetramer.

Loops A, C, and E form highly ordered stable structures within the tetramer that are unexpectedly constant and unaltered by bound Fabs. This accounts for the lack of Fab binding to linear peptides of the AQP4 loop epitopes alone since peptides could suffer entropic cost to conform to the structured conformations displayed on APQ4 (*11*, *12*). In addition, in OAPs, any of three loops from three or four different monomers may contribute in different orientations to the epitope resulting in a more complex assembly than for a tetramer alone. Hence, a diagnostic peptide binding assay to detect serum AQP4-IgG might need to retain some of the ordered displayed epitope. Furthermore, AQP4 OAP formation adds to epitope complexity, as the extracellular loops on multiple arrayed AQP4 tetramers are presented in close proximity.

Lattices of membrane proteins in physiology are rare. While there are 13 different AQPs in human physiology, AQP0 from the eye lens is the only other case of aquaporin arrays (*26*–*28*). The lattices of AQP0 are formed from protein-protein contacts that are completely different from the lipid-mediated interfaces in AQP4. The unit cell dimension in AQP4 arrays is *a* = 69 Å (*18*) versus *a* = 65.5 Å in AQP0 arrays. Thus, the area of the unit cell in AQP4 corresponds to ~8 more lipids per tetramer. In AQP4 OAPs, the tetramers are rotated relative to those of AQP0 to form protein-protein contacts between tetramers, opening a larger lipid-filled area between four tetramers (fig. S14). While there is recognized physiological rationale for AQP0 lattices (*29*), the AQP4 M23 lattices are structurally unrelated and have not yet any recognized physiological reason for their presence.

Given the prominent role of AQP4-IgG in NMO lesion pathogenesis (*1*–*3*), direct inhibition of antibody binding would minimize damage and neurologic impairment. Interaction between AQP4-IgG and AQP4 may modulate both antibody effector function and the local inflammatory milieu, thereby modifying glial and neuronal injury (*8*, *30*). The 3D structures of rAbs and AQP4 epitopes define interfaces that lead to assembly of antibody platforms that facilitate CDC. AQP4 rAbs sensitive to C loop mutations at H151/L154 enhance complement C1q activation on AQP4 OAPs by facilitating assembly of hexameric antibody complexes on AQP4 OAPs (*8*, *31*). The two atomic structures of Fab58 and Fab186-bound AQP4 provide a template that can instruct the design of small molecule, peptide, or protein inhibitors that might block serum AQP4-IgG binding by directly competing with antibody binding or altering the structure of the epitopes on AQP4. Previous attempts to select inhibitory compounds have not been informed by a blueprint of the direct interactions between AQP4-IgG and AQP4 (*32*). These structures demonstrate the intricacy of composite epitope recognition and provide a platform for intelligent inhibitor design.

## MATERIALS AND METHODS

### AQP4 expression and purification

The full-length human AQP4 gene isoform M1 (AQP4 M1) was synthesized and cloned in pPICZ expression vector (GenScript). The expression construct was designed with an N-terminal 8xHis followed by a flag tag (DYKDDDDK) and a thrombin cleavage site and cloned into the EcoR1 and Not1 sites of the vector. A protocol similar to previously published was used for the expression of AQP4 M1 in *Pichia pastoris* with modifications (*14*). In summary, the expression vector was electroporated into *P. pastoris* X-33 cells and the transformed cells were then selected as colonies on yeast extract peptone dextrose plates with Zeocin (50 g/ml; Invitrogen) and tested for expression. More than 100 colonies were screened in batches and compared to the best AQP4 expressor of each round to yield a high-expression stable strain of *P. pastoris* (MG3). For protein production, the *P. pastoris* MG3 strain was cultured in growth media at 30°C for 24 hours, then the temperature was lowered to 26°C, and methanol was added directly to the cultures to a final concentration of 2.5%. The cultures were grown for another 48 hours. Cultures were harvested by centrifugation at 4°C and 6000*g* for 10 min. Cells were resuspended in the lysis buffer [1× tris-buffered saline with 2 mM 2-mercaptoethanol (βME)] and lysed by bead beating with glass beads. Broken and unlysed cells were removed by centrifugation at 4°C and 6000*g* for 10 min. The supernatant was pelleted at 160,000*g* at 4°C for 2 hours to extract the membranes. Pellets were resuspended in MR buffer [25 mM tris HCl (pH 7.4) at room temperature, 250 mM NaCl, 10% glycerol, and 1 mM βME] and stored at −80°C until further processing. To begin purification, resuspended membranes were solubilized by adding 1% *n*-dodecyl-β-d-maltoside (DDM) (Anatrace) to a final concentration of 200 mM and stirred at 4°C for 1 hour. Unsolubilized material was pelleted at 160,000*g* at 4°C for 30 min. Imidazole (50 mM, pH 7.4) was added to the supernatant. The supernatant was then batch bound with Ni–nitrilotriacetic resins (Qiagen) for 2 hours, loaded onto a Bio-Rad Econo Column and washed with MR buffer with 30 mM βDDM and 50 mM imidazole, and then eluted with 300 mM imidazole. Imidazole was removed using Econo-Pac DG10 desalting column (Bio-Rad) equilibrated with MR buffer with 30 mM βDDM. The N-terminal tag was cleaved by thrombin at 4°C overnight. Uncleaved AQP4 was removed the next day with HisPur Cobalt Resin (Thermo Fisher Scientific). Then, AQP4 was concentrated in a 50,000 molecular weight cutoff Amicon spin concentrator (Millipore) and further purified by size exclusion chromatography on a Superdex 200 Increase 10/300 GL column (Cytiva) in 25 mM citrate (pH 6.0), 50 mM NaCl, 5% glycerol, 30 mM βDDM, and 2 mM dithiothreitol.

### AQP4 nanodisc reconstitution

Purified AQP4 in detergent with intact affinity tags was used for this reconstitution. AQP4 (100 μM) was mixed with 300 μM purified 1E3D1 MSP and 10 mM Soy Polar Lipids (Avanti). The mixture was incubated overnight at 4°C with Bio-Beads SM2 resin (Bio-Rad) to adsorb the detergent from the solution. The sample was separated from Bio-Beads and allowed to bind to HisPur Cobalt Resin (Thermo Scientific) to separate reconstituted nanodiscs with AQP4 and remove empty nanodiscs using affinity tag on AQP4. Reconstituted AQP4 in nanodiscs was then eluted with 150 mM imidazole–containing buffer. The elute was concentrated using 50,000-kDa molecular weight cutoff Amicon spin concentrator (Millipore) and further purified by size exclusion chromatography on a Superose 6 Increase 10/300 GL column (Cytiva) in 50 mM HEPES (pH 7.5) and 100 mM NaCl buffer.

### rAb expression and purification

Recombinant monoclonal NMO antibodies rAb58 and rAb186 were generated as described (*5*, *16*) from clonally expanded cerebrospinal fluid plasmablasts obtained from an AQP4-IgG–seropositive patient following informed consent (COMIRB 00-688). VH and VL constructs were cotransfected (1:3 ratio) into Expi293F cells for expression. The supernatant was harvested, centrifuged to remove any cells and debris, adjusted to pH 8, and purified at 4°C on an AKTA pure 25 system using a HiTrap MabSelect affinity column and HiPrep 26/10 desalting column (Cytiva). The rAb was eluted from the MabSelect column in 0.1 M glycine (pH 2.7) and desalted using 1× phosphate-buffered saline (PBS). Desalted rAb was adjusted to pH 7.5 with 1 M tris-HCl (pH 8.0) and exchanged and concentrated in PBS using Ultracel YM-30 microconcentrators (Millipore). Antibody integrity and Fab fragments were confirmed by denaturing and native–polyacrylamide gel electrophoresis (PAGE), and IgG concentration was assayed using a Nanodrop spectrophotometer (Thermo Fisher Scientific).

### Fab preparation

A Pierce Fab purification kit (Thermo Scientific) was used to make Fabs from the IgGs following the manufacturer’s protocol. In summary, papain cleavage was performed to digest human IgGs, resulting in Fab and Fc fragments. The Fc fragment was separated using Protein A agarose. The purity of Fab was assessed on SDS-PAGE gel.

### Biolayer interferometry

The MSP 1E3D1 was biotinylated before AQP4 nanodisc reconstitution. Then AQP4 in biotinylated MSP 1E3D1 was immobilized on Octet BLI Streptavidin Biosensor tips (Sartorius). Patient-derived recombinant IgGs were kept in solution at various concentrations. Octet Red 384 system was used at 25°C with 1000-rpm shaking for the measurements. The IgG binding was assayed using BLI on the immobilized AQP4 tips using a 16-channel experiment. Dissociation coefficients (*K*_d_) were estimated assuming heterogenous ligand fitting model. An IgG isotype control was used to assess the baseline of the experiment.

### Cryo-EM sample preparation

AQP4 M1 isoforms in 1E3D1 nanodiscs were used at 0.3 μM for the apo structure. For AQP4-Fab58 sample, AQP4 in nanodiscs was mixed with ~5-fold molar excess of Fab58. AQP4-Fab186 sample was prepared in a similar way. Final concentration of AQP4 in the mixture was 0.3 μM at the time of grid freezing in all the samples. A mixed population of cryo-EM 2D classes containing 1 to 4 Fab186-bound AQP4 tetramers was obtained, and hence an increased Fab186 ratio to 30-fold molar excess that of AQP4 was used. This resulted in saturation of all four binding sites on the AQP4 tetramer. There was a severe orientation bias with this sample that we were not able to rectify even after using multiple data collection and processing strategies. This prohibited us from achieving a high-resolution map. AQP4 in detergent was used for obtaining the Fab186-bound structure where 18 μM AQP4 was mixed with ~75 μM Fab186. Final AQP4 (in detergent) concentration on grids was ~12 μM.

For grid preparation, Mark IV Vitrobot (FEI) was used at 10°C and 100% humidity. Three microliters of the sample was applied to freshly glow-discharged Quantifoil R 1.2/1.3 400-mesh Au holey carbon grids. Blotting time used was between 3 and 7 s with a blot force of −2 to remove excess sample from the grids. Rapid plunge freezing was done in liquid ethane after blotting and grids were stored in liquid nitrogen.

### Cryo-EM data collection

Cryo-EM data acquisition was on Titan Krios, a 300-kV transmission electron microscope, at a defocus range from −0.8 to −2.5 and a total dose between 45 and 60 e/Å^2^. The Titan Krios was operated with a postcolumn energy filter (20 eV slit width) and GATAN K3 direct electron detector. The data collection was setup using Serial-EM with automated data acquisition features. Number of micrographs collected for each structure, dose, and pixel size are listed in table S2. For AQP4-Fab58 structure, two datasets, one at 0° stage tilt and another at 30° stage tilt, were collected. Addition of 30° tilt data helped us overcome the orientation bias problem. For AQP4-Fab186 structure, the datasets were collected at 0° stage tilt and 20° stage tilt for the same reason.

### Cryo-EM data processing and model building

The movies obtained from data collection were subjected to dose-weighted beam-induced motion correction using MotionCor2 to obtain the micrographs. CryoSPARC (versions 3.2 and 4.2.1) was used for further data processing (*33*). In brief, the Patch CTF in CryoSPARC was used for CTF estimation and the micrographs were manually curated. The 0° and 30° stage tilt data were merged for Fab58 after this step. Similarly, 0° and 20° stage tilt data were merged for Fab186 data. Blob picker was used for particle picking to generate templates for eventual template picking of the particles. After particles picking, 2D classification was performed. It was followed by ab initio 3D volume generation and heterogenous classification using ab initio volumes. Multiple rounds of 2D classification, ab initio volume generation, and heterogenous classification were performed. The best-quality map from these steps was used for nonuniform refinement. C4 symmetry was imposed for the AQP4 apo structure, while AQP4-Fab58 and AQP4-Fab186 structures were determined without any imposed symmetry during data processing.

For model building of AQP4 part of the structures, we used our previously published crystal structure model as the starting point [Protein Data Bank (PDB): 3GD8] (*14*). We used Coot for model building and refinements in Phenix, software hosted by SBGrid consortium (*34*). For the Fab fragments, AlphaFold model was first generated using the known amino acid sequences (*35*). The predicted model was then refined in Coot and Phenix according to the cryo-EM map obtained.

### OAP estimation

Hiroaki *et al.* (*18*) determined the structure of the rat M23 isoform of AQP4 that forms a double bilayer assembly in which the external faces of AQP4 tetramers in each layer associate to form a double bilayer. Their 3D structure was determined by electron diffraction to a resolution of 3.2 Å in plane/3.6 Å perpendicular to the plane of the double bilayers (*18*) (fig. S14). This association (PDB: ID 2D57) resulted in deformation of the external loops as compared to our previously published crystal structure of AQP4 and cryo-EM structures in this article (*14*). The cell dimensions in OAPs are identical to those of the double layer, and may have a role, arguably though not proven in cell-cell adhesion. One layer of the double bilayer interface was used as the template for OAPs (fig. S15). We therefore superimposed our experimentally determined AQP4 tetramer structure into each unit in the array (fig. S15A). The rat and human AQP4 arrays have the same cell dimensions, the same as found for OAPs, and their sequences are highly conserved (fig. S15B).

To assess if Fab186 can potentially interact with a neighboring tetramer in an OAP, we used minimal unit of Fab186 interaction from our structures, one Fab186 molecule and two monomers from a tetramer. From our array estimation we extracted two adjacent tetramers. The minimal AQP4-Fab186 interaction unit was imposed on the two adjacent array tetramers (Fig. 5).
