# Supplementary material for: Structural basis of aquaporin-4 autoantibody binding in neuromyelitis optica
Source: Sci Adv. 2025 Feb 21;11(8):eadq7560. doi: 10.1126/sciadv.adq7560 (PMC11844742; doi:10.1126/sciadv.adq7560)
Supplement: Supplementary file 1 — Figs. S1 to S16 Tables S1 and S2 Legend for movie S1 [file sciadv.adq7560_sm.pdf]

Science Advances
AAAS

Supplementary Materials for

Structural basis of aquaporin-4 autoantibody binding in neuromyelitis optica

Meghna Gupta *et al.*

Corresponding author: Meghna Gupta, guptame@ohsu.edu; Jeffrey L. Bennett, jeffrey.bennett@cuanschutz.edu; Robert M. Stroud, stroud@msg.ucsf.edu

Sci. Adv. **11**, eadq7560 (2025)
DOI: 10.1126/sciadv.adq7560

The PDF file includes:

Figs. S1 to S16
Tables S1 and S2

Other Supplementary Material for this manuscript includes the following:

Movie S1

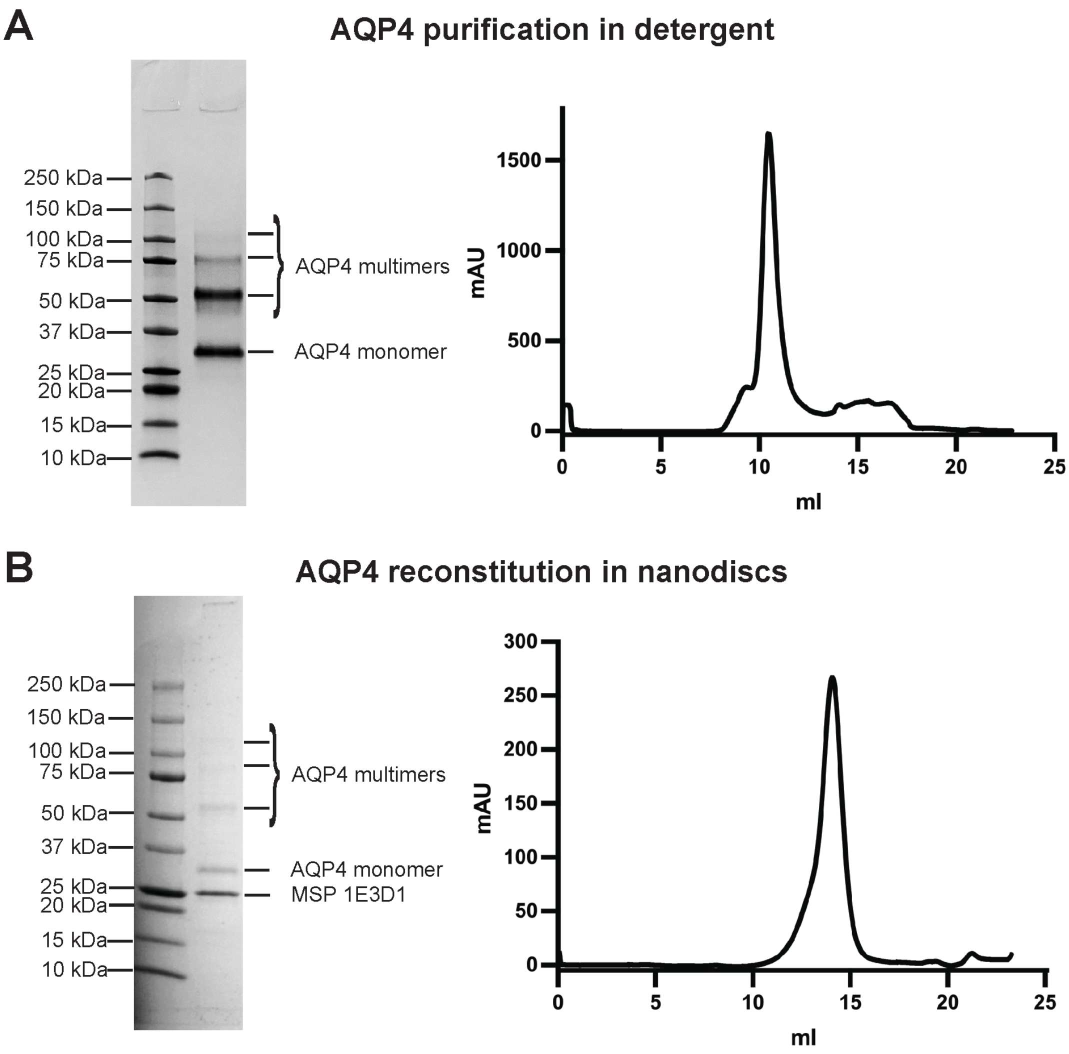

Fig. S1.
Human AQP4 purification and nanodisc reconstitution. (A) Purified AQP4 in detergent analyzed on SDS-PAGE and SEC profile of AQP4 in detergent using Superdex™ 200 Increase 10/300 GL column. **(B)** Reconstitution of purified AQP4 in lipid nanodiscs- SDS-PAGE and SEC profile using Superose™ 6 Increase 10/300 GL column.

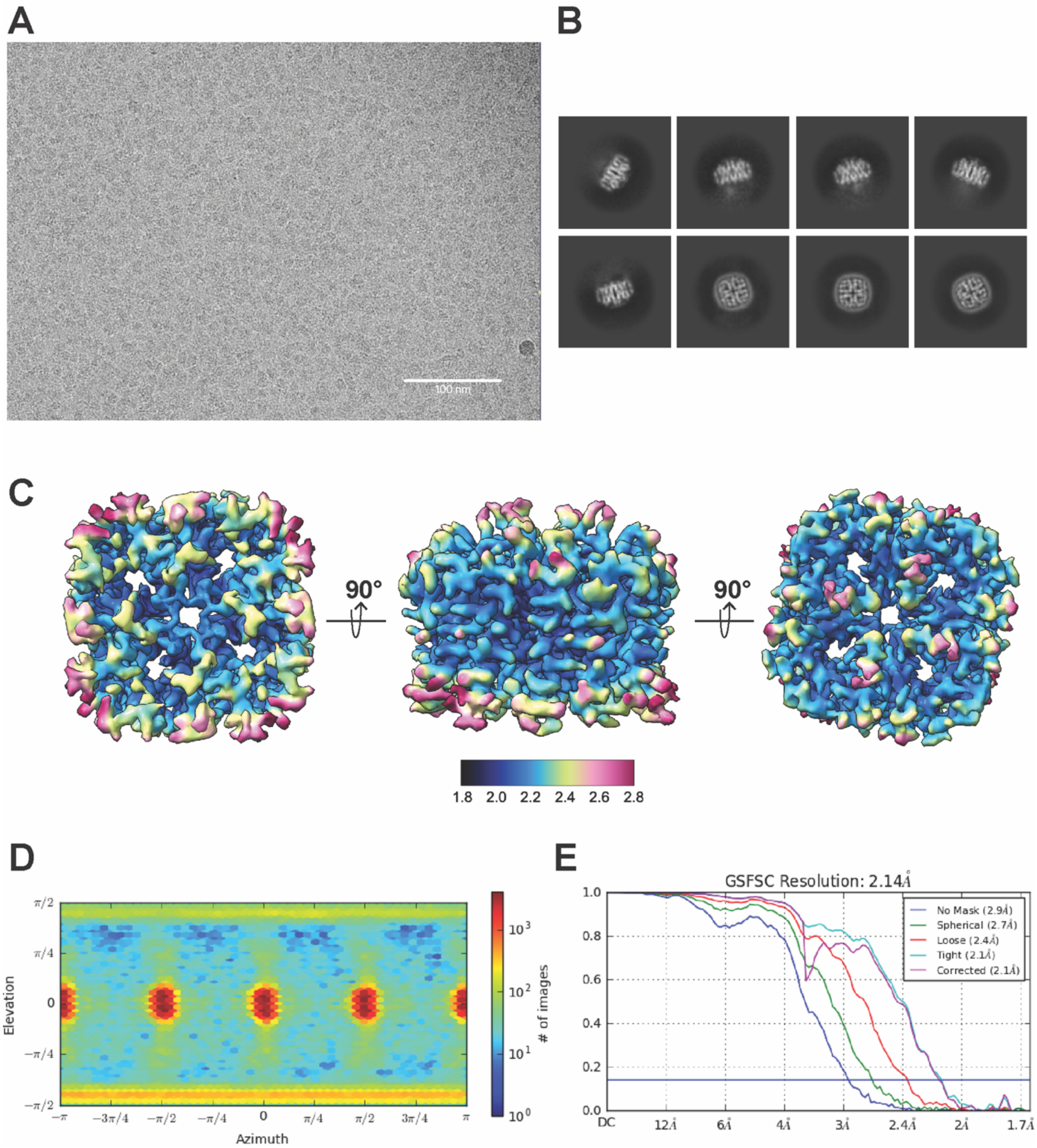

Fig. S2.
AQP4 apo cryoEM data collection and processing. (A) Representative micrograph from cryoEM experiment after motion correction. **(B)** Representative 2D classes from cryoEM data processing. **(C)** The top, side and bottom view of the final map obtained from the cryoEM data processing. **(D)** Euler angle distribution of the particle images. **(E)** Fourier shell correlation (FSC) curves showing gold standard resolution at 0.143.

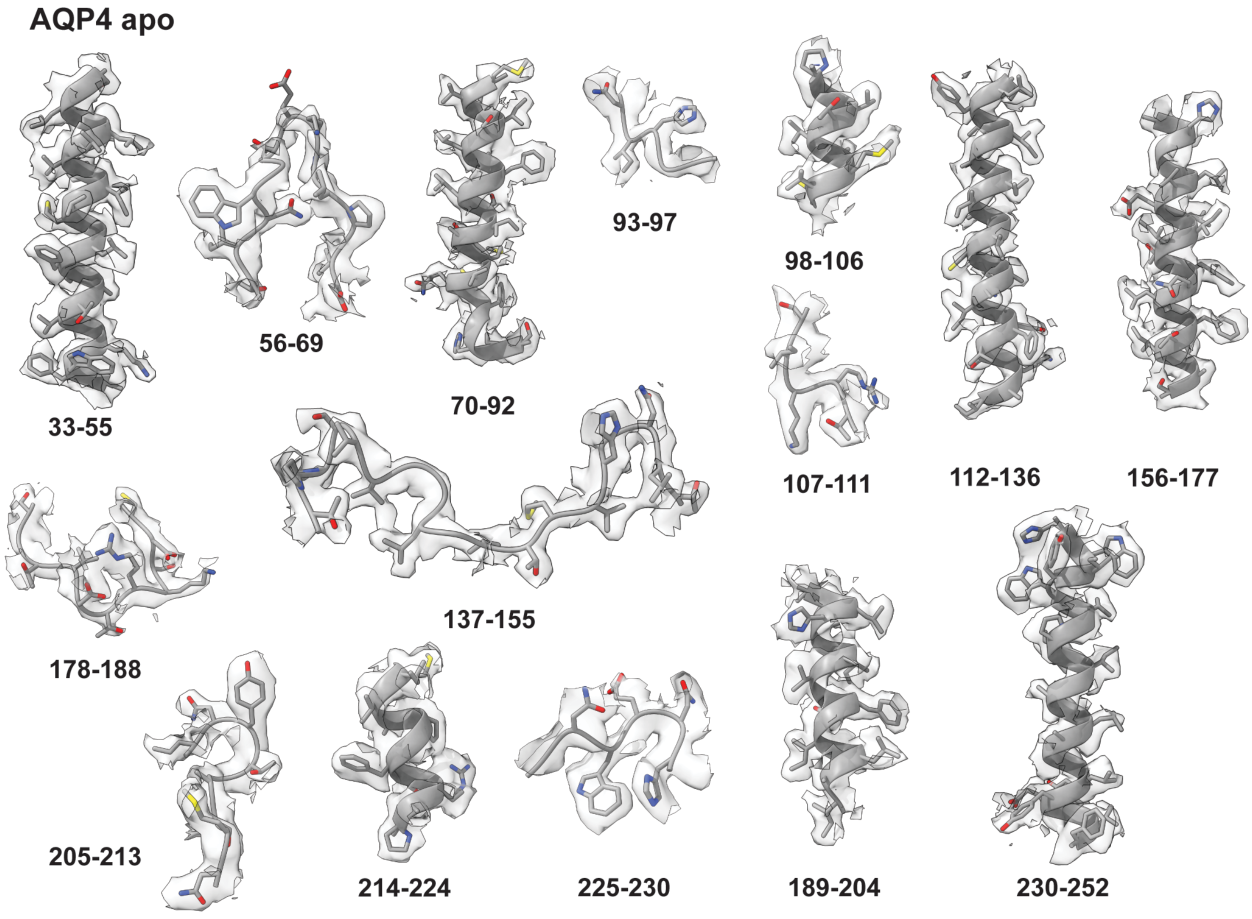

Fig. S3.
AQP4 apo representative densities. EM densities for the transmembrane helices and loops for the AQP4 apo with the model built into it.

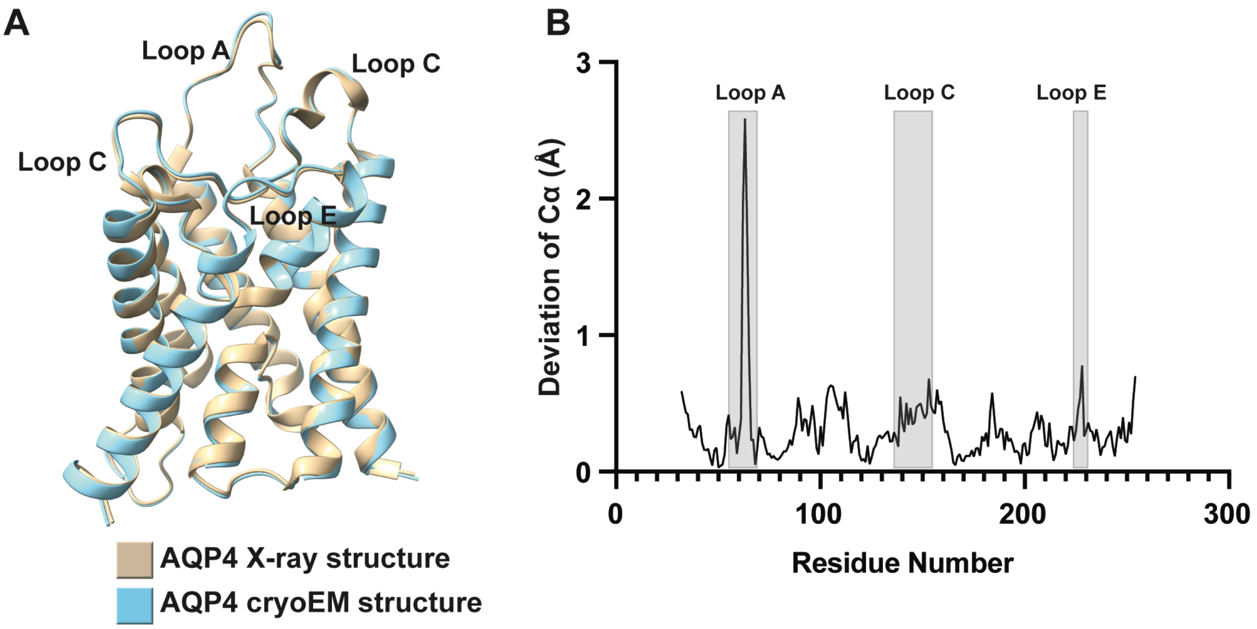

Fig. S4.
Comparison between AQP4 structure obtained using X-ray crystallography and cryoEM.
(A) Overlay of a monomer of AQP4 from X-ray crystallography (PDB: 3GD8) in tan and cryoEM structures in cyan. **(B)** Representation of deviation in Cα rmsd per residue comparing X-ray crystallography and cryoEM structures. The difference in loop A is an artifact due to poor density in the X-ray structure for the extremity of two residues.

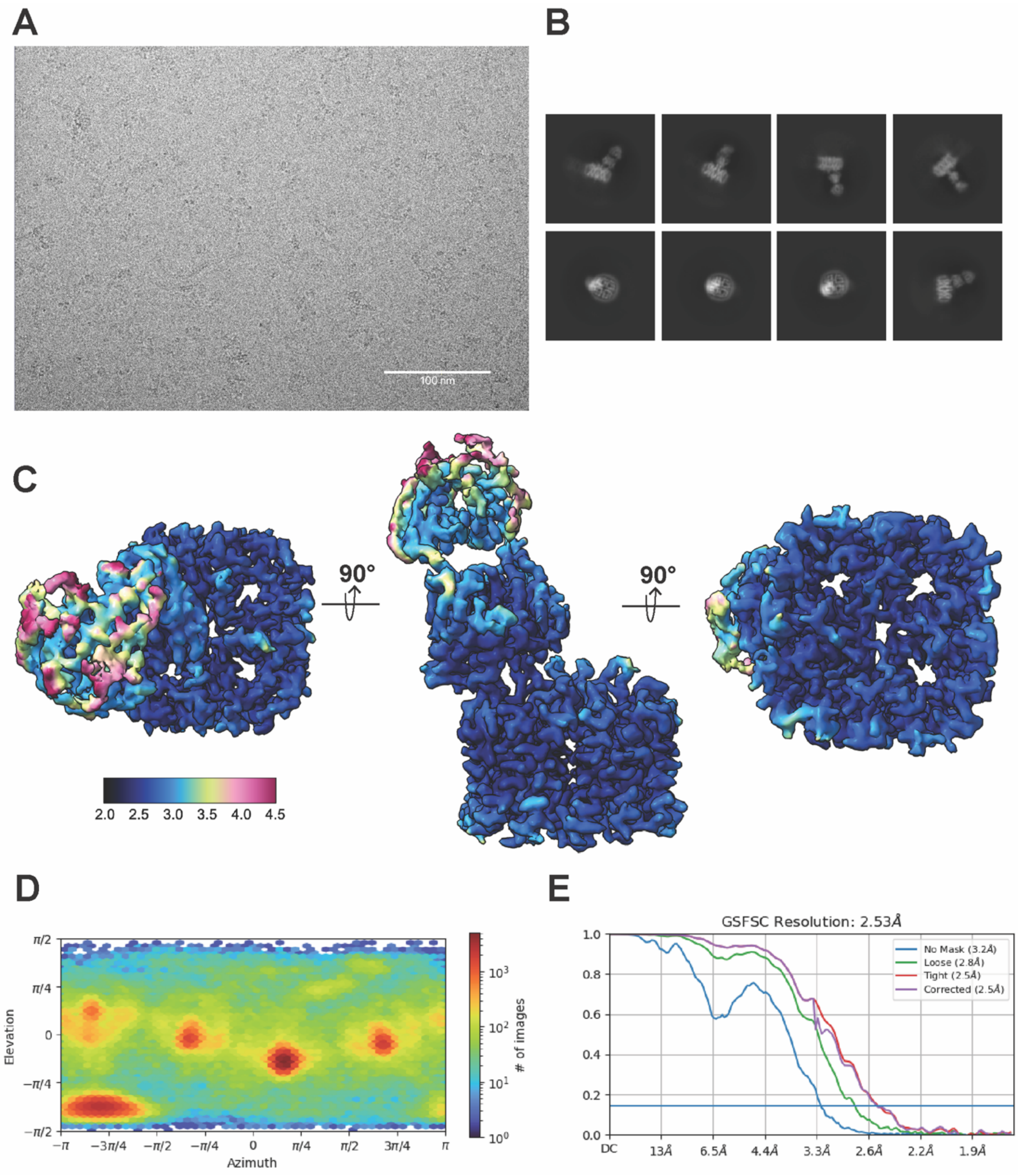

Fig. S5.

AQP4-Fab58 cryoEM data collection and processing. (A) Representative micrograph from cryoEM experiment after motion correction. **(B)** Representative 2D classes from cryoEM data processing. **(C)** The top, side and bottom view of the final map obtained from the cryoEM data processing. **(D)** Euler angle distribution of the particle images. **(E)** FSC curves showing gold standard resolution at 0.143.

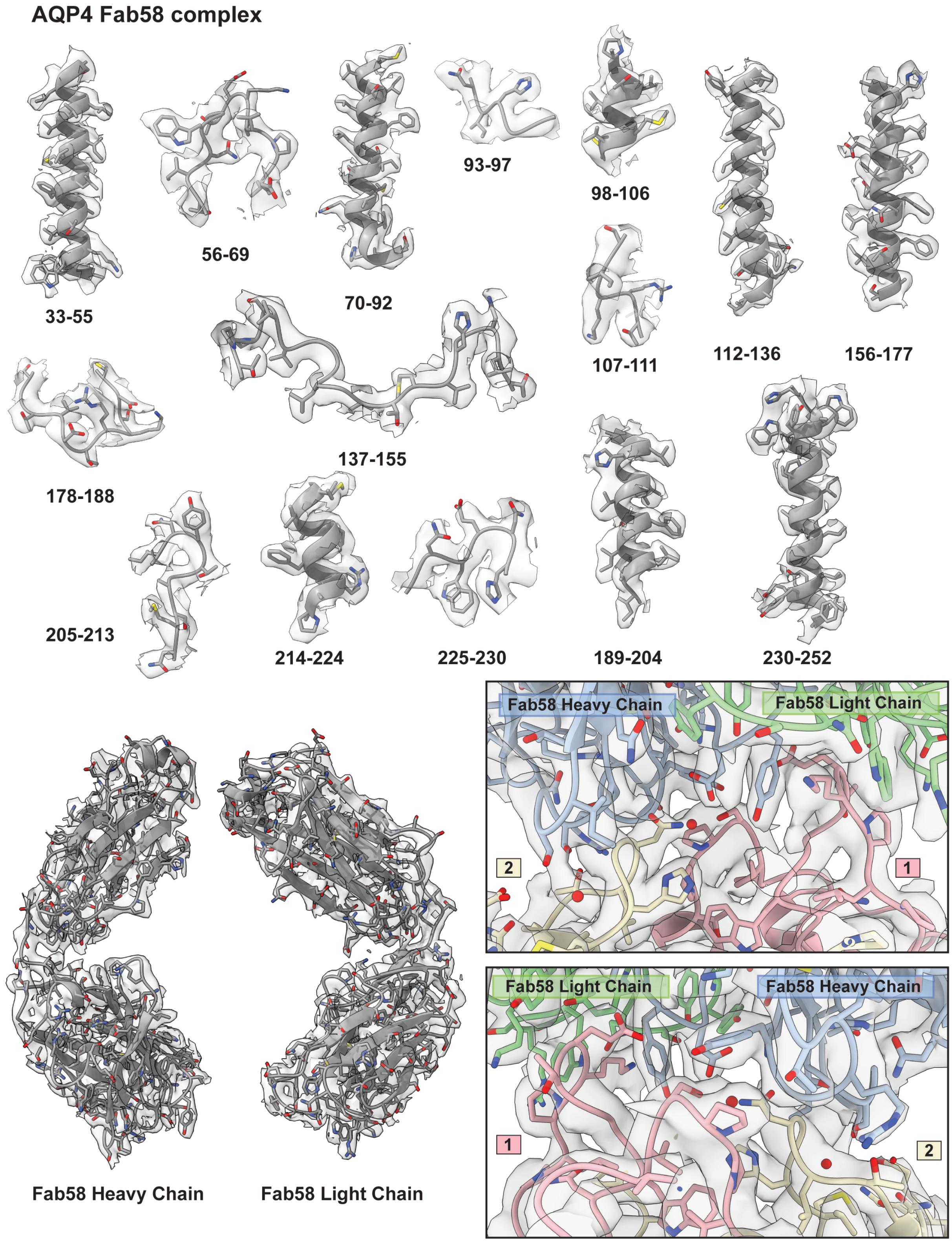

Fig. S6.
AQP4-Fab58 representative densities. EM densities for the transmembrane helices and loops for the AQP4-Fab58 with the model built into it.

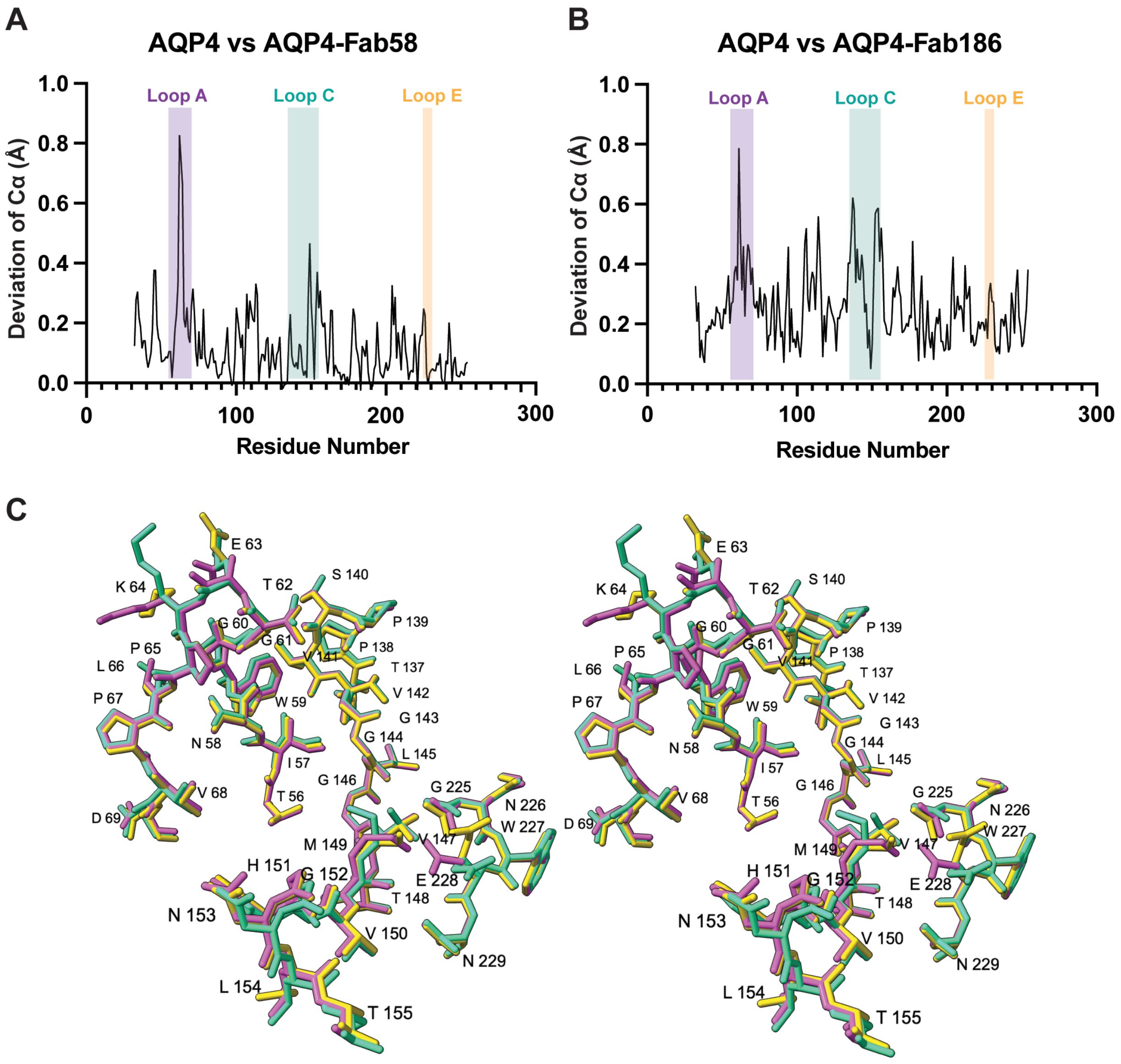

Fig. S7.

Differences in AQP4 loops on Fab binding. Representation of deviation in Cα rmsd per residue comparing AQP4 apo and Fab bound structures- **(A)** AQP4 vs AQP4-Fab58, **(B)** AQP4 vs AQP4-Fab186. **(C)** The loops A, C, and E are displayed in a crossed eyed stereoscopic image. The structure in yellow is for AQP4 apo (yellow) viewed obliquely from outside the membrane to separate the chains such that loop A is on the left, loop C down the center of the figure, loop E to the right side. The structure of chains when attached by Fab58 (pink), and Fab186 (green) are shown to illustrate the differences in epitopes versus the unbound structure.

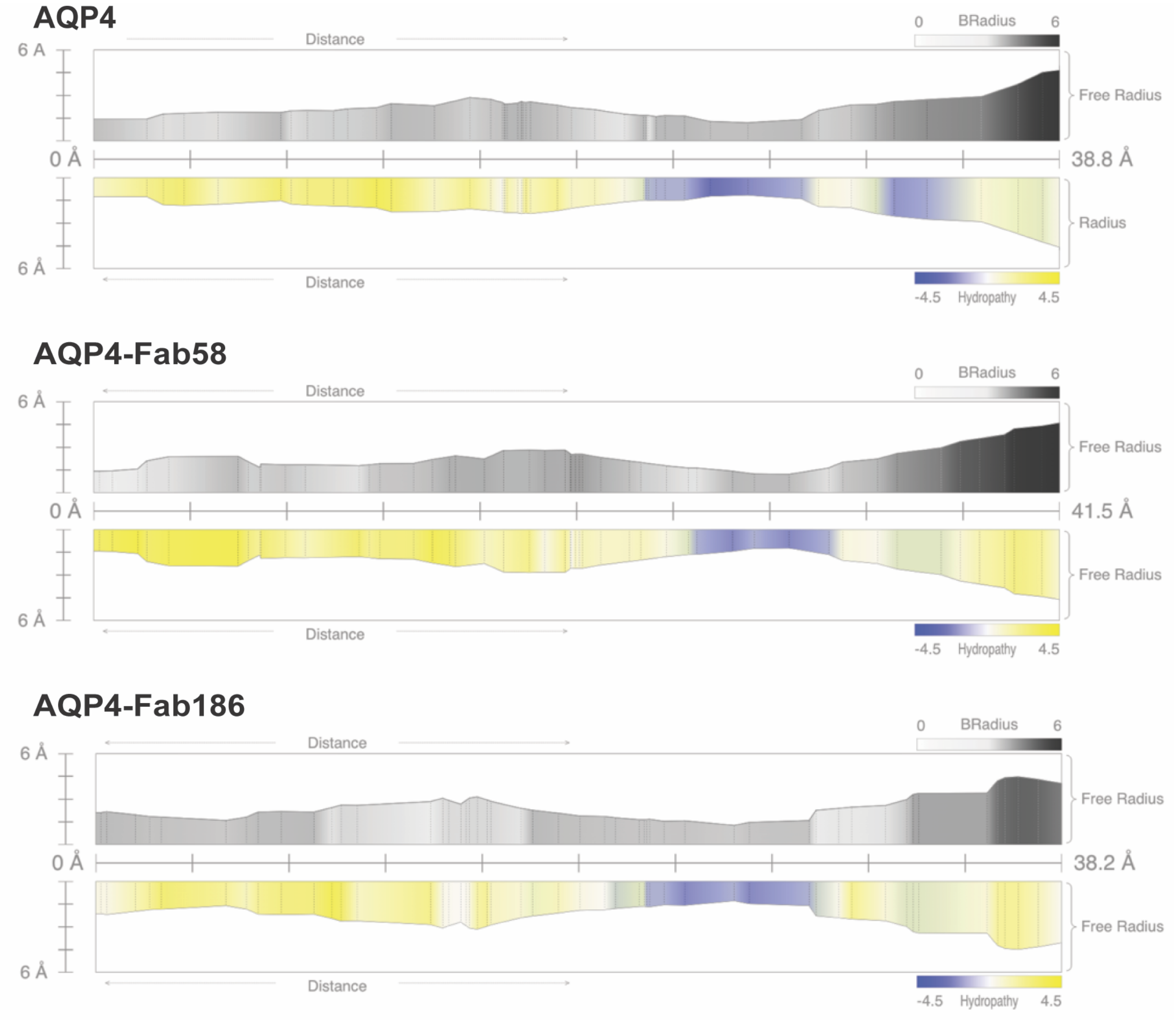

Fig. S8.
Assessment of any restriction on the water channel in the apo and Fab bound structures.
Diameter of water channel across AQP4 using HOLE (*17*) program for AQP4, AQP4-Fab58, and AQP4-Fab186 structures showing constancy of water channel in AQP4 upon Fab binding.

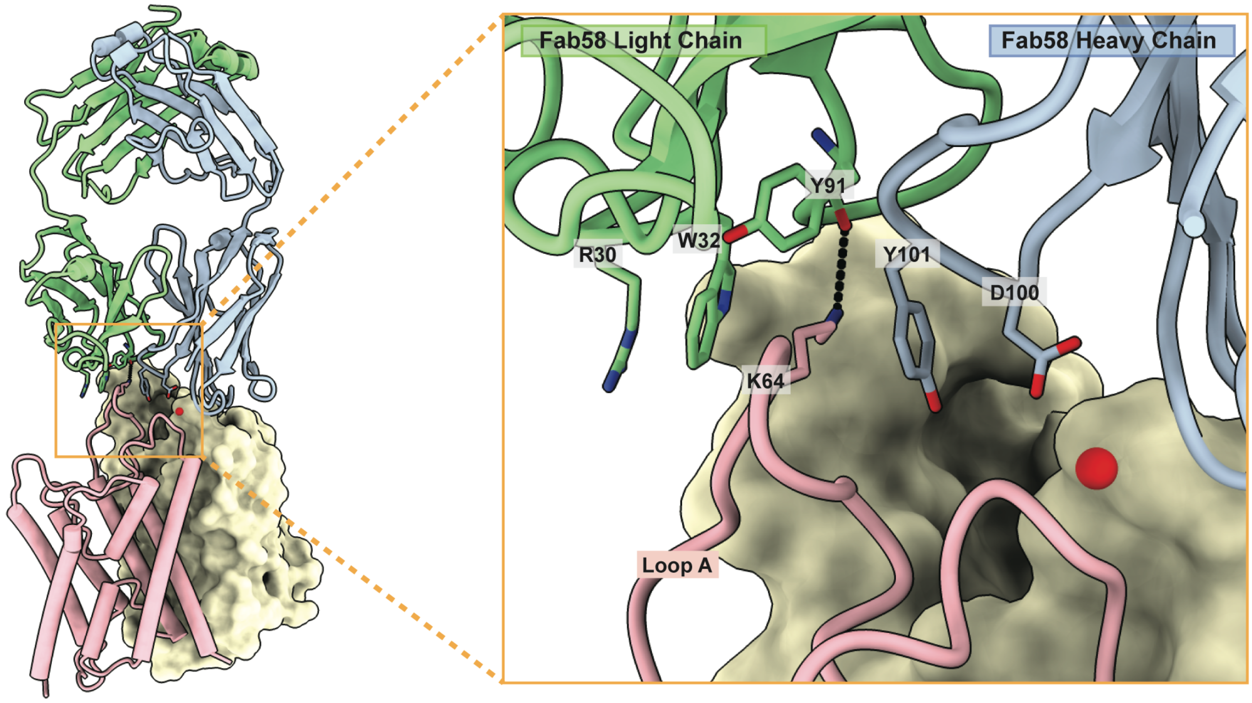

Fig. S9.
AQP4-Fab58 loop A interactions. Interactions of loop A with HC and LC of Fab58.

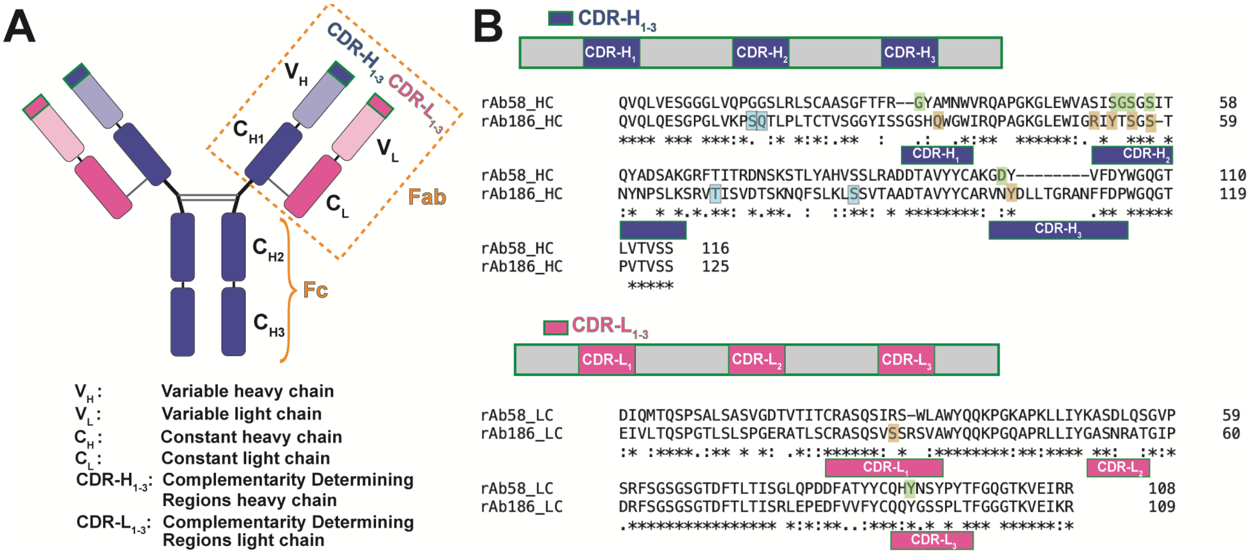

Fig. S10.

Overall IgG structure and mapping CDRs on the Fab58 and Fab186. (A) Cartoon representing the domains of the IgG antibody type. Box with dotted line highlights the Fab region of the antibody used for structure determination. **(B)** Sequence alignment of heavy and light chains Fab58 and Fab186 are shown in top and bottom part of the figure, respectively. The CDR regions (1-3) in both heavy and light chains are marked for both the Fabs. AQP4 interacting residues in Fab58 are highlighted in green while Fab186 residues are highlighted in orange. Predicted lateral interaction residues in Fab186 with AQP4 OAPs are highlighted in cyan.

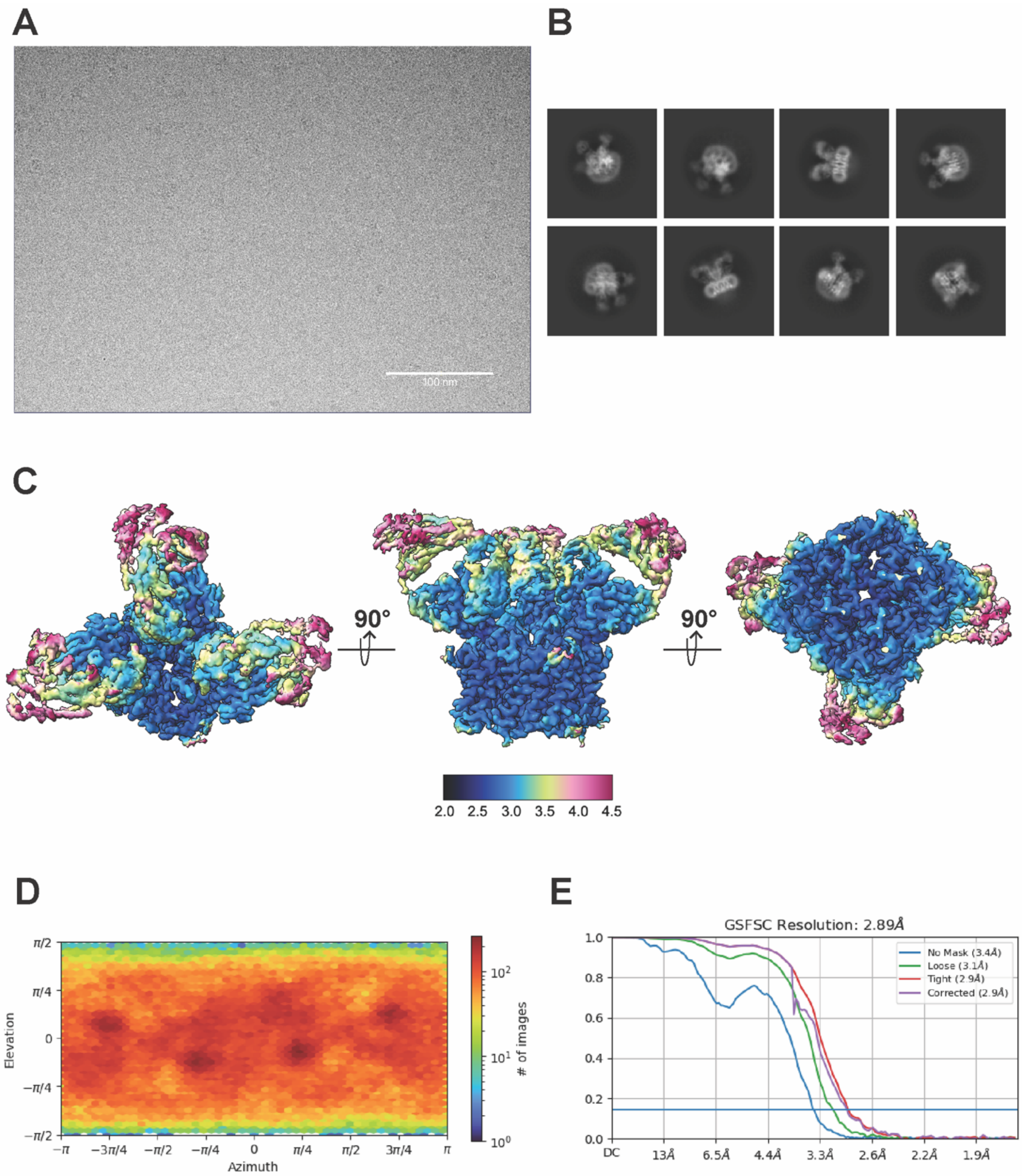

Fig. S11.
AQP4-Fab186 cryoEM data collection and processing. (A) Representative micrograph from cryoEM experiment after motion correction. **(B)** Representative 2D classes from cryoEM data processing. **(C)** The top, side and bottom view of the final map obtained from the cryoEM data processing. **(D)** Euler angle distribution of the particle images. **(E)** FSC curves showing gold standard resolution at 0.143.

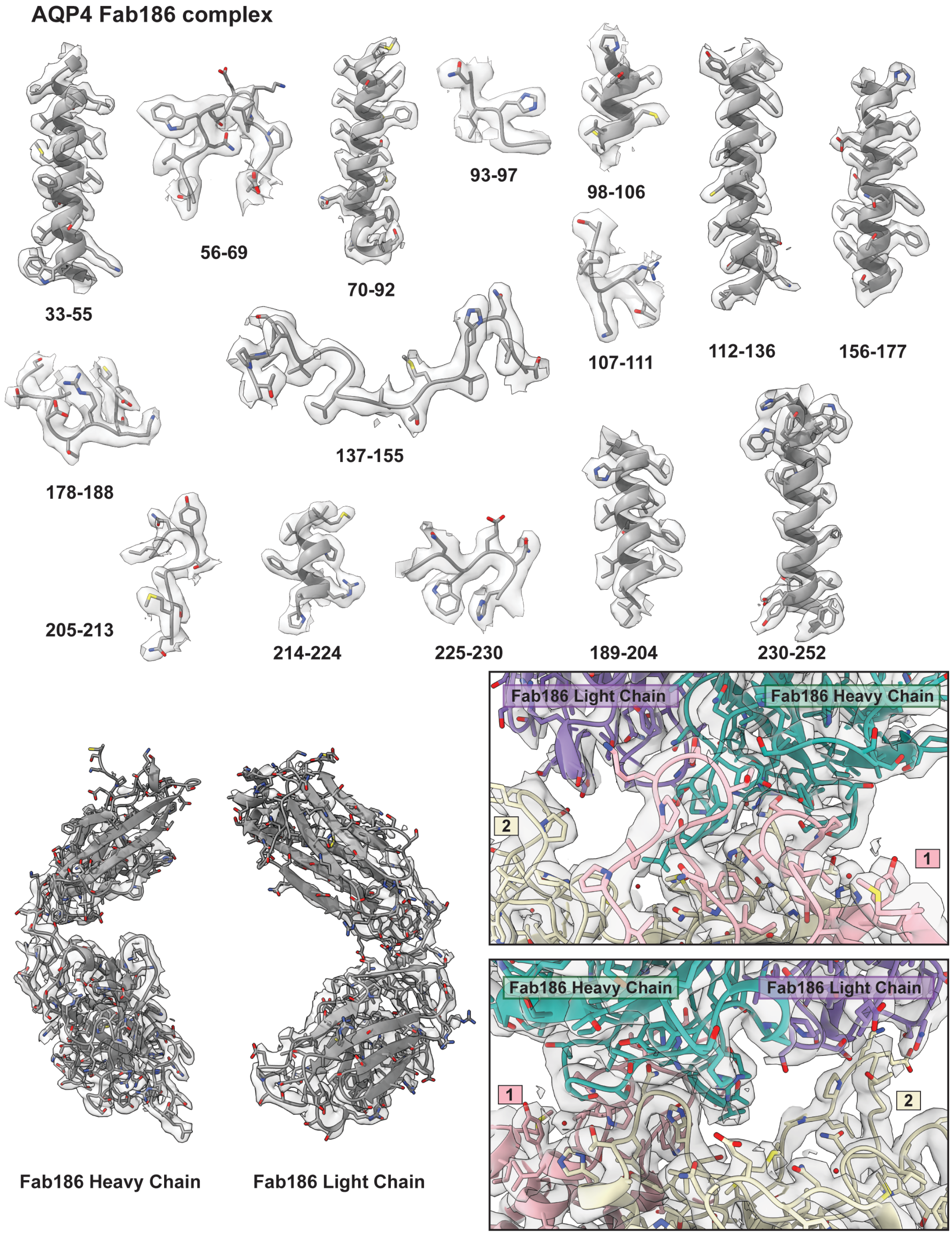

Fig. S12.
AQP4-Fab186 representative densities. EM densities for the transmembrane helices and loops for the AQP4-Fab186 with the model built into it.

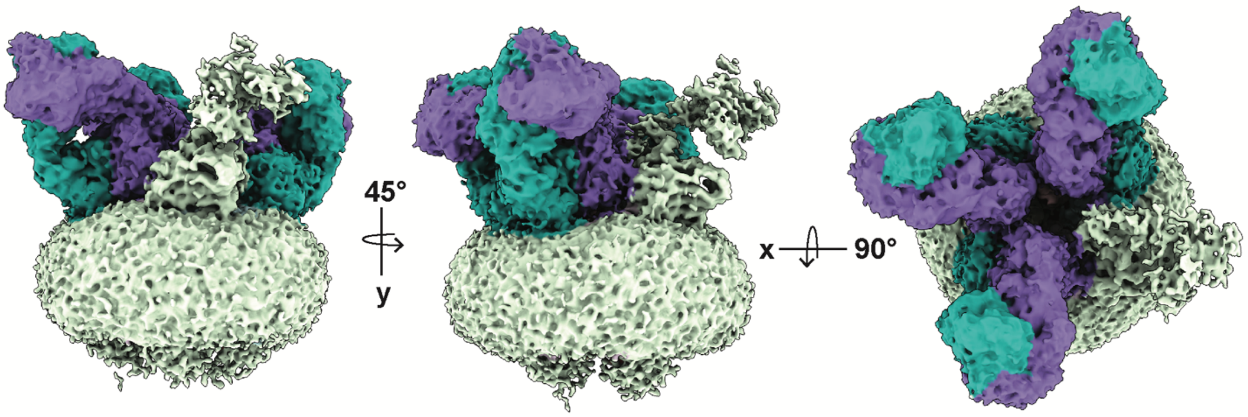

Fig. S13.
AQP4-Fab186 low contour map exhibiting the density for a fourth Fab186. The final cryoEM map shows densities prominently for three Fab186 bound to the tetramer but there is a weak density for the fourth Fab molecule. The fourth density is clearly visible in the 2D classes as well.

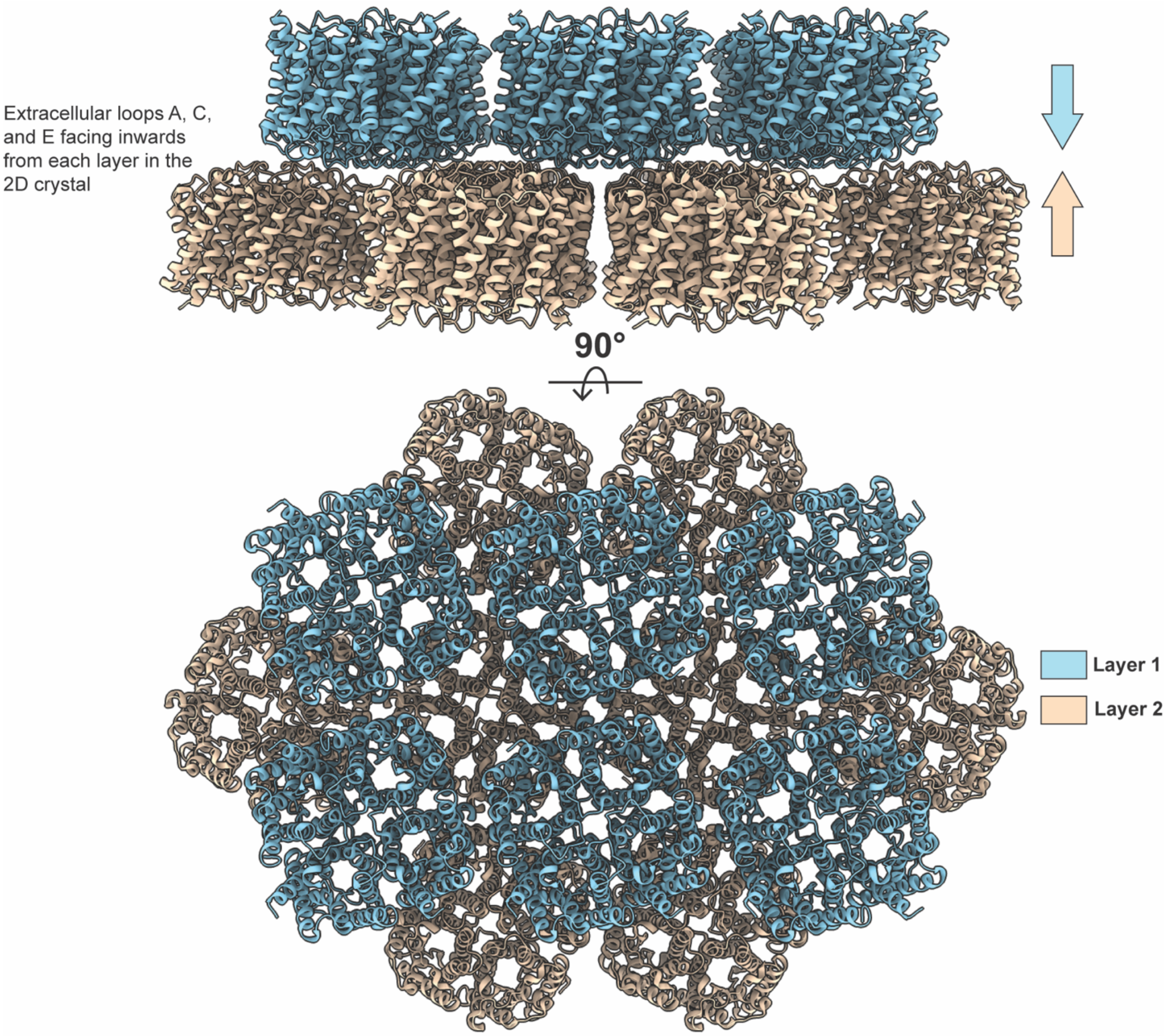

Fig. S14.

Representation of two AQP4 layers in the 2D crystal. Side view and top view of the 2D electron diffraction structure of rat AQP4 M23 isoform (PDB ID 2D57) exhibiting two layers of AQP4 array with the extracellular loops facing inwards. Layer 1 in color sky blue and Layer 2 in color peach.

A

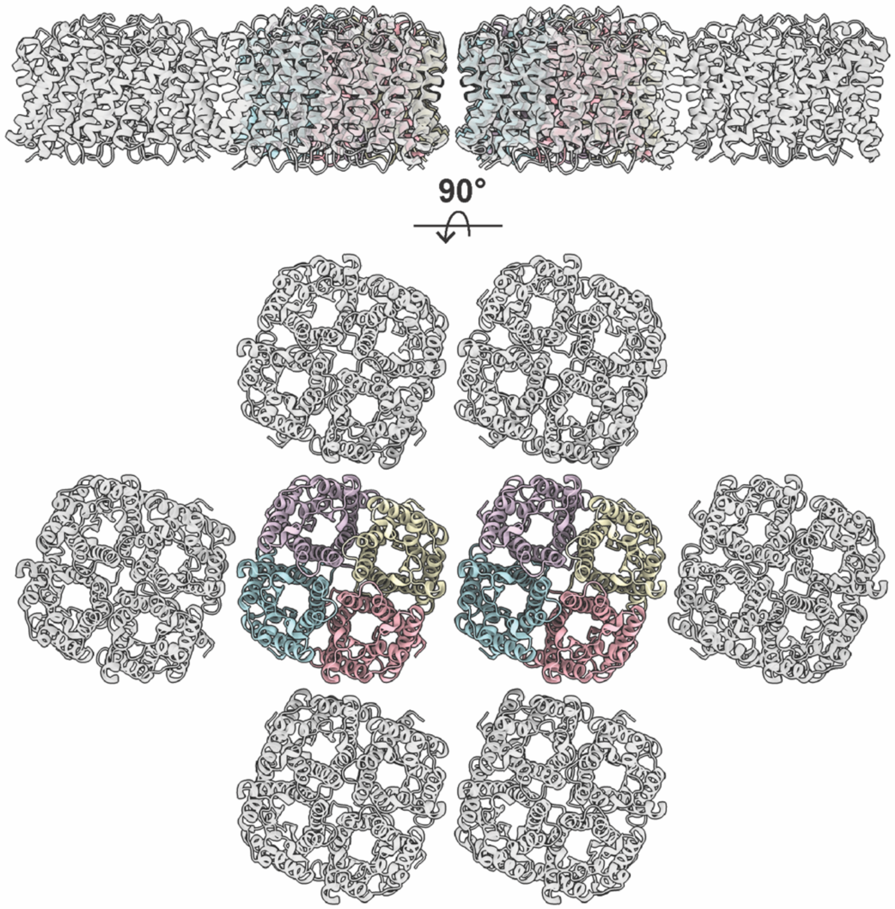

B

```
AQP4_HUMAN      MSDRPTARRWGKCGPLCTRENIMVAFKGVWTQAFWKAVTAEFLAMLIFVLLSLGSTINWG    60
AQP4_RAT        MSDGAAARRWGKCGPPCSRESIMVAFKGVWTQAFWKAVTAEFLAMLIFVLLSVGSTINWG    60
                ***  :********* *:**.******************************:********

AQP4_HUMAN      GTEKPLPVDMVLISLCFGLSIATMVQCFGHISGGHINPAVTVAMVCTRKISIAKSVFYIA    120
AQP4_RAT        GSENPLPVDMVLISLCFGLSIATMVQCFGHISGGHINPAVTVAMVCTRKISIAKSVFYIT    120
                *:*:*******************************************************:

AQP4_HUMAN      AQCLGAIIGAGILYLVTPPSVVGGLGVTMVHGNLTAGHGLLVELIITFQLVFTIFASCDS    180
AQP4_RAT        AQCLGAIIGAGILYLVTPPSVVGGLGVTTVHGNLTAGHGLLVELIITFQLVFTIFASCDS    180
                **************************** *******************************

AQP4_HUMAN      KRTDVTGSIALAIGFSVAIGHLFAINYTGASMNPARSFGPAVIMGNWENHWIYWVGPIIG    240
AQP4_RAT        KRTDVTGSVALAIGFSVAIGHLFAINYTGASMNPARSFGPAVIMGNWENHWIYWVGPIIG    240
                ********:***************************************************

AQP4_HUMAN      AVLAGGLYEYVFCPDVEFKRRFKEAFSKAAQQTKGSYMEVEDNRSQVETDDLILKPGVVH    300
AQP4_RAT        AVLAGALYEYVFCPDVELKRRLKEAFSKAAQQTKGSYMEVEDNRSQVETEDLILKPGVVH    300
                *****.***********:***:***************************:**********

AQP4_HUMAN      VIDVDRGEEKKGKDQSGEVLSSV 323
AQP4_RAT        VIDIDRGDEKKGKDSSGEVLSSV 323
                ***:***:******.********
```

Fig. S15.

Estimation of spatial arrangement of AQP4 in OAPs. (A) Side view and top view of the single layer of AQP4 array derived from 2D lattice. For the two representative neighboring tetramers same color scheme as in other figures was used. **(B)** Sequence comparison of Human and Rat AQP4.

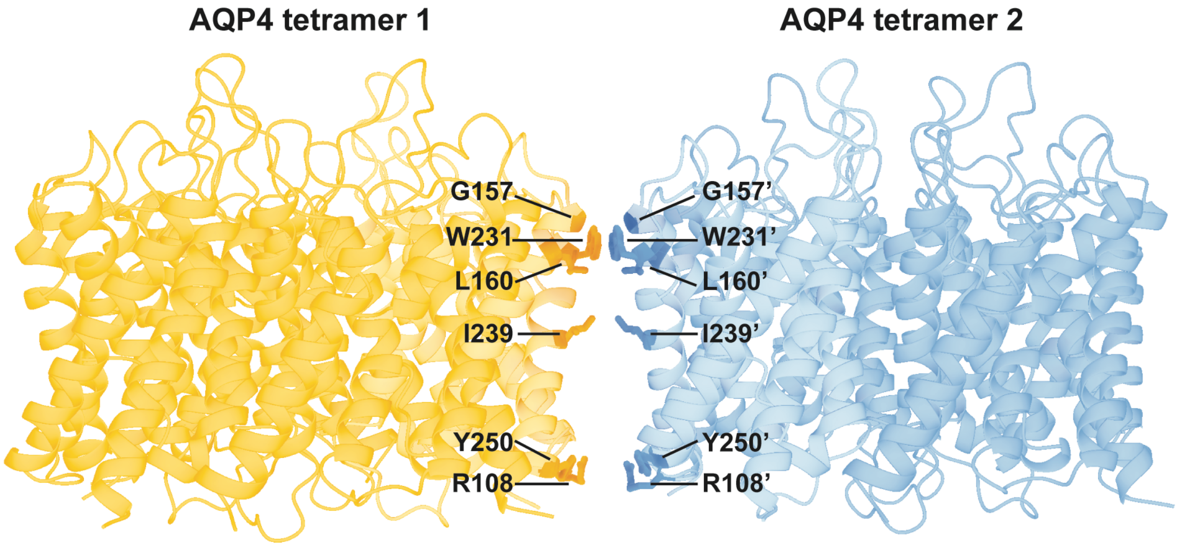

Fig. S16.
AQP4 inter-tetramer interface in OAPs. Two AQP4 tetramers modelled against OAP are shown here with amino acid side chains highlighted that are known to stabilize the inter-tetramer interaction (*18*).

AQP4 apo cryoEM vs X-ray diffraction structures	
Overall Cα rmsd mean	0.304Å
Loop A Cα rmsd	0.674Å
Loop C Cα rmsd	0.413Å
Loop E Cα rmsd	0.439Å
**AQP4-Fab58 vs AQP4 apo cryoEM structures**	
Overall Cα rmsd mean	0.171Å
Loop A Cα rmsd	0.347Å
Loop C Cα rmsd	0.201Å
Loop E Cα rmsd	0.148Å
**AQP4-Fab186 vs AQP4 apo cryoEM structures**	
Overall Cα rmsd mean	0.254Å
Loop A Cα rmsd	0.397Å
Loop C Cα rmsd	0.374Å
Loop E Cα rmsd	0.244Å

Table S1.

Rmsd comparison of AQP4 (cryoEM vs X-ray diffraction), and AQP4 apo vs Fab bound structures.

	**AQP4 Apo**<br>(EMD-43044)<br>(PDB 8V8S)	**AQP4-Fab58**<br>(EMD-43047)<br>(PDB 8V91)	**AQP4-Fab186**<br>(EMD-43071)<br>(PDB 8V9D)
**Data collection and processing**			
Magnification	105,000x	105,000x	105,000x
Voltage (kV)	300	300	300
Electron exposure (e–/Å^2)	~60	~60	~46
Defocus range (μm)	0.8-2.0	0.8-2.0	0.8-2.0
Pixel size (Å)	0.834	0.834	0.834
Symmetry imposed	C4	C1	C1
Initial particle images (no.)	1,839,188	4,072,284	7,279,511
Final particle images (no.)	371,328	323,342	220,335
Map resolution (Å)<br>(FSC=0.143)	2.1	2.5	2.9
**Refinement**			
Model-to-map CC_{mask}	0.78	0.79	0.87
Model-to-map CC_{box}	0.63	0.70	0.72
Model-to-map CC_{peaks}	0.59	0.60	0.70
Model-to-map CC_{volume}	0.78	0.78	0.82
Model composition<br>Non-hydrogen atoms<br>Protein residues<br>Ligands	 6717<br>892<br>0	 9915<br>1320<br>0	 16683<br>2221<br>0
B factors (Å^2)<br>Iso/Aniso (#)<br>Protein (min/max/mean)	 6717/0<br>27.34/104.51/62.71	 9915/0<br>67.00/163.39/91.21	 16683/0<br>0.49/115.44/42.53
Bonds (RMSD)<br>Length (Å) (# > 4σ)<br>Angles (°) (# > 4σ)	 0.002 (0)<br>0.472 (0)	 0.004 (0)<br>0.969 (0)	 0.004 (0)<br>0.964 (0)
Validation<br>MolProbity score<br>Clashscore<br>Rotamers outliers (%)	 1.55<br>6.43<br>0.00	 1.61<br>8.68<br>0.00	 1.51<br>6.00<br>0.00
Ramachandran plot<br>Favored (%)<br>Allowed (%)<br>Disallowed (%)	 96.83<br>3.17<br>0.00	 97.24<br>2.76<br>0.00	 96.96<br>3.04<br>0.00

Table S2.
CryoEM data collection and processing statistics.

Movie S1.

Morph between Fab58 and Fab186 on two AQP4 monomers. Both Fab58 and Fab186 interactions span over two monomers within a AQP4 tetramer. To make a comparison of their interaction with respect to AQP4, this linear morph represents transition from Fab58 binding to Fab186 binding over two monomers.
